# Supplementary material for: An intranasal vaccine durably protects against SARS-CoV-2 variants in mice
Source: Cell Rep. 2021 Jul 10;36(4):109452. doi: 10.1016/j.celrep.2021.109452 (PMC8270739; doi:10.1016/j.celrep.2021.109452)
Supplement: Document S1. Figures S1 and S2 [file mmc1.pdf]

Cell Reports, Volume *36*

Supplemental information

An intranasal vaccine durably protects against SARS-CoV-2 variants in mice

Ahmed O. Hassan, Swathi Shrihari, Matthew J. Gorman, Baoling Ying, Dansu Yaun, Saravanan Raju, Rita E. Chen, Igor P. Dmitriev, Elena Kashentseva, Lucas J. Adams, Colin Mann, Meredith E. Davis-Gardner, Mehul S. Suthar, Pei-Yong Shi, Erica Ollmann Saphire, Daved H. Fremont, David T. Curiel, Galit Alter, and Michael S. Diamond

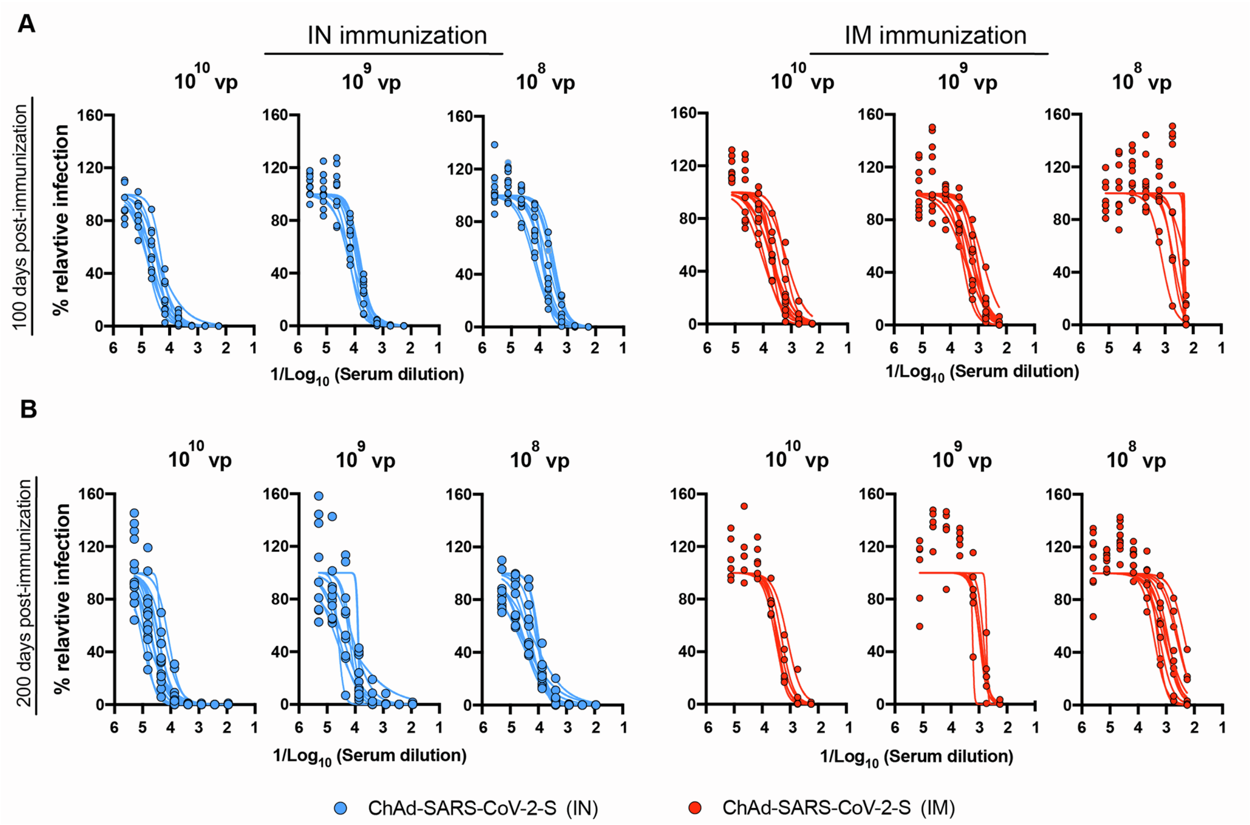

Figure S1. ChAd-SARS-CoV-2-S vaccine induces neutralizing antibodies as measured by FRNT. Related to Figure 1. Five-week old female BALB/c mice were immunized via IN or IM route with a single 10^{10}, 10^9, or 10^8 dose of ChAd-SARS-CoV-2-S. Serum samples from ChAd-SARS-CoV-2-S vaccinated mice were collected at days 100 (**A**) or 200 (**B**) after immunization and assayed for neutralizing activity by FRNT. Serum neutralization curves corresponding to individual mice are shown for the indicated vaccines (n = 6-14 per group). Each point represents the mean of two technical replicates.

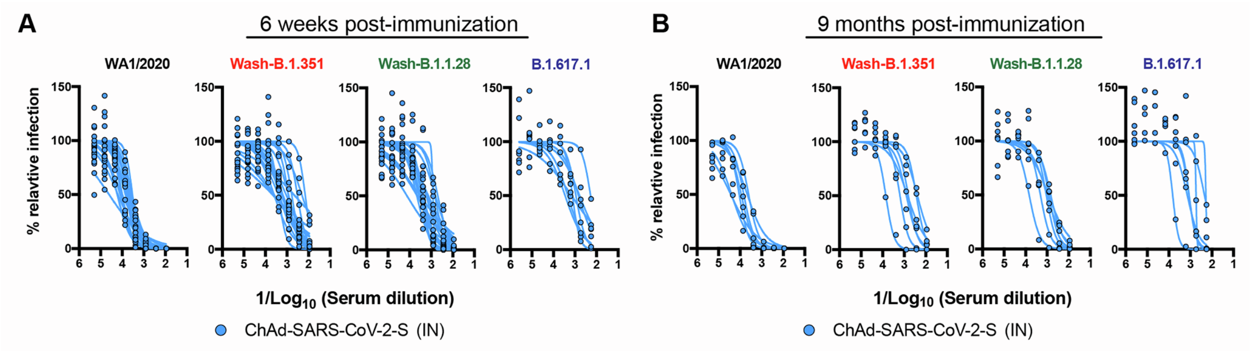

Figure S2. ChAd-SARS-CoV-2-S vaccine induces neutralizing antibodies as measured by FRNT. Related to Figure 4. Five-week-old K18-hACE2 female mice were immunized with 10^9 vp ChAd-control or ChAd-SARS-CoV-2-S via an IN route. Serum samples were collected at six weeks (**A**) or nine months (**B**) after immunization and assayed for neutralizing activity against WA1/2020, Wash-B.1.351, Wash-B.1.1.28, or B.1.617.1 by FRNT. Serum neutralization curves corresponding to individual mice are shown for the indicated vaccines (n = 7-20 per group). Each point represents the mean of two technical replicates.
