# Supplementary material for: An intranasal vaccine durably protects against SARS-CoV-2 variants in mice
Source: Cell Rep. 2021 Jul 10;36(4):109452. doi: 10.1016/j.celrep.2021.109452 (PMC8270739; doi:10.1016/j.celrep.2021.109452)
Supplement: Document S2. Article plus supplemental information [file mmc2.pdf]

Cell Reports

Article

An intranasal vaccine durably protects against SARS-CoV-2 variants in mice

Graphical abstract

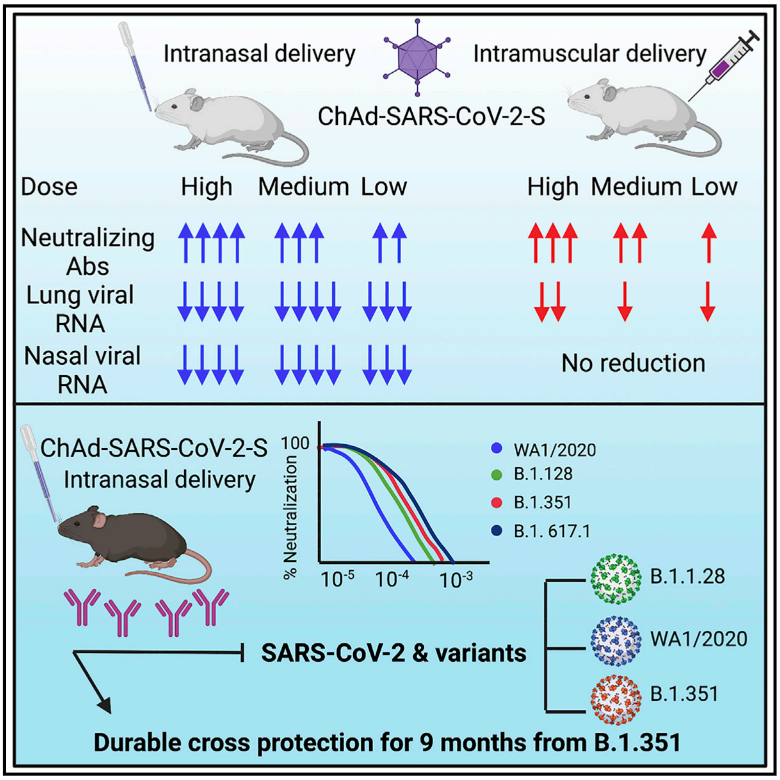

Highlights

- Immunization with ChAd-SARS-CoV-2 S induces durable immunity
- Intranasal ChAd-SARS-CoV-2 S induces inhibitory IgG and IgA Abs
- Abs induced by intranasal ChAd-SARS-CoV-2 S have robust Fc effector functions
- Intranasal ChAd-SARS-CoV-2 S confers cross-protection against variants of concern

Authors

Ahmed O. Hassan, Swathi Shrihari, Matthew J. Gorman, ..., David T. Curiel, Galit Alter, Michael S. Diamond

Correspondence

diamond@wusm.wustl.edu

In brief

Hassan et al. show that immunization with ChAd-SARS-CoV-2-S is durably immunogenic and protects against SARS-CoV-2 challenge in a dose-dependent manner. Many months after single-dose intranasal immunization, ChAd-SARS-CoV-2 confers protection against variants of concerns of SARS-CoV-2 in both the upper and lower respiratory tracts of mice.

Hassan et al., 2021, Cell Reports *36*, 109452
July 27, 2021 © 2021 The Author(s).
https://doi.org/10.1016/j.celrep.2021.109452

CellPress

Cell Reports

CellPress
OPEN ACCESS

Article

An intranasal vaccine durably protects against SARS-CoV-2 variants in mice

Ahmed O. Hassan,[1] Swathi Shrihari,[1] Matthew J. Gorman,[2] Baoling Ying,[1] Dansu Yaun,[2] Saravanan Raju,[1,3] Rita E. Chen,[1,3] Igor P. Dmitriev,[4] Elena Kashentseva,[4] Lucas J. Adams,[3] Colin Mann,[5] Meredith E. Davis-Gardner,[6] Mehul S. Suthar,[6] Pei-Yong Shi,[7] Erica Ollmann Saphire,[5] Daved H. Fremont,[3,8,9] David T. Curiel,[4] Galit Alter,[2] and Michael S. Diamond[1,3,9,10,11,*]

[1]Department of Medicine, Washington University School of Medicine, St. Louis, MO 63110, USA
[2]Ragon Institute of Massachusetts General Hospital (MGH), Massachusetts Institute of Technology, and Harvard University, Cambridge, MA, USA
[3]Department of Pathology & Immunology, Washington University School of Medicine, St. Louis, MO 63110, USA
[4]Department of Radiation Oncology, Washington University School of Medicine, St. Louis, MO 63110, USA
[5]La Jolla Institute for Immunology, La Jolla, CA 92037, USA
[6]Center for Childhood Infections and Vaccines of Children's Healthcare of Atlanta, Department of Pediatrics, Emory Vaccine Center, Emory University School of Medicine, Atlanta, GA 30322, USA
[7]Department of Biochemistry and Molecular Biology, University of Texas Medical Branch, Departments of Microbiology and Immunology, University of Texas Medical Branch, Sealy Institute for Vaccine Sciences, University of Texas Medical Branch, Galveston, TX 77555, USA
[8]Department of Biochemistry and Molecular Biophysics, Washington University School of Medicine, St. Louis, MO 63110, USA
[9]Department of Molecular Microbiology, Washington University School of Medicine, St. Louis, MO 63110, USA
[10]The Andrew M. and Jane M. Bursky Center for Human Immunology and Immunotherapy Programs, Washington University School of Medicine, St. Louis, MO 63110, USA
[11]Lead contact
*Correspondence: diamond@wusm.wustl.edu
https://doi.org/10.1016/j.celrep.2021.109452

SUMMARY

SARS-CoV-2 variants that attenuate antibody neutralization could jeopardize vaccine efficacy. We recently reported the protective activity of an intranasally administered spike protein-based chimpanzee adenovirus-vectored vaccine (ChAd-SARS-CoV-2-S) in animals, which has advanced to human trials. Here, we assessed its durability, dose response, and cross-protective activity in mice. A single intranasal dose of ChAd-SARS-CoV-2-S induced durably high neutralizing and Fc effector antibody responses in serum and S-specific IgG and IgA secreting long-lived plasma cells in the bone marrow. Protection against a historical SARS-CoV-2 strain was observed across a 100-fold vaccine dose range and over a 200-day period. At 6 weeks or 9 months after vaccination, serum antibodies neutralized SARS-CoV-2 strains with B.1.351, B.1.1.28, and B.1.617.1 spike proteins and conferred almost complete protection in the upper and lower respiratory tracts after challenge with variant viruses. Thus, in mice, intranasal immunization with ChAd-SARS-CoV-2-S provides durable protection against historical and emerging SARS-CoV-2 strains.

INTRODUCTION

Severe acute respiratory syndrome coronavirus 2 (SARS-CoV-2) is the etiologic agent of the coronavirus disease 2019 (COVID-19) syndrome, which can rapidly progress to pneumonia, respiratory failure, and systemic inflammatory disease (Cheung et al., 2020; Mao et al., 2020; Wichmann et al., 2020). The elderly, immunocompromised, and those with certain co-morbidities (e.g., obesity, diabetes, and hypertension) are at greatest risk of severe disease, requirement of mechanical ventilation, and death (Zhou et al., 2020). To date, approximately 184 million infections and 4 million deaths have been recorded worldwide (https://covid19.who.int) since the start of the pandemic. The extensive morbidity and mortality associated with COVID-19 pandemic have made the development and deployment of SARS-CoV-2 vaccines an urgent global health priority.

The spike (S) protein of the SARS-CoV-2 virion is the principal target for antibody-based and vaccine countermeasures. The S protein serves as the primary viral attachment and entry factor and engages the cell-surface receptor angiotensin-converting enzyme 2 (ACE2) to promote SARS-CoV-2 entry into human cells (Letko et al., 2020). SARS-CoV-2 S proteins are cleaved to yield S1 and S2 fragments (Hoffmann et al., 2020), with the S1 protein containing the receptor binding domain (RBD) and the S2 protein promoting membrane fusion and virus penetration into the cytoplasm. The prefusion form of the SARS-CoV-2 S protein (Wrapp et al., 2020) is recognized by potently neutralizing monoclonal antibodies (Barnes et al., 2020; Cao et al., 2020b; Pinto et al.,

This is an open access article under the CC BY license (http://creativecommons.org/licenses/by/4.0/).

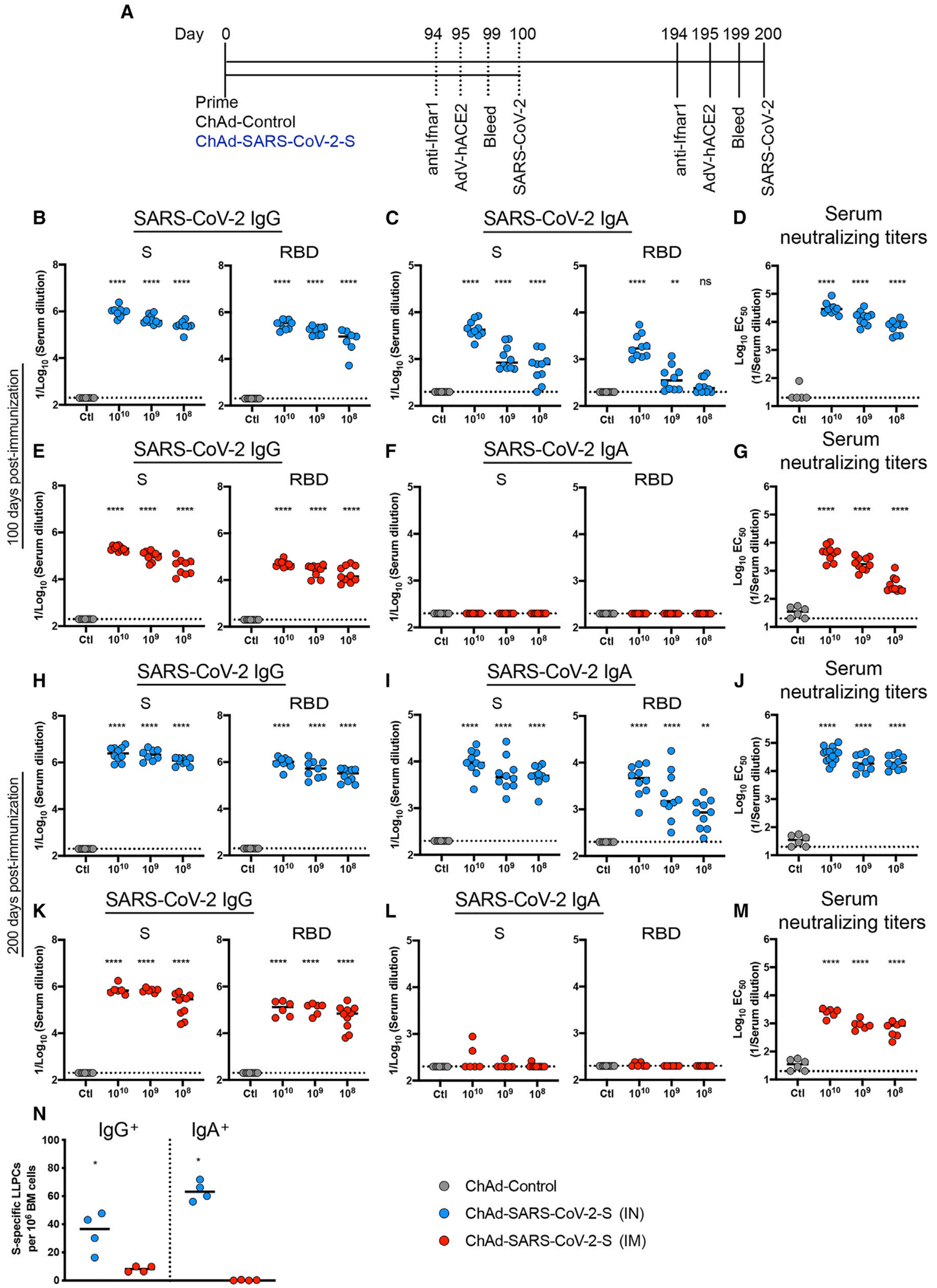

(legend on next page)

2020; Tortorici et al., 2020; Zost et al., 2020) or protein inhibitors (Cao et al., 2020a).

Many vaccine candidates targeting the SARS-CoV-2 S protein have been developed (Burton and Walker, 2020) using DNA plasmid, lipid nanoparticle encapsulated mRNA, inactivated virion, protein subunit, or viral-vectored vaccine platforms (Graham, 2020). Several vaccines administered by intramuscular (IM) injection (e.g., Pfizer/BioNTech BNT162b2 and Moderna-1273 mRNA [Baden et al., 2021; Polack et al., 2020] and Johnson & Johnson Ad26.COV2 and AstraZeneca ChAdOx1 nCoV-19 adenoviral [Barrett et al., 2021; Sadoff et al., 2021]) platforms) have been granted emergency-use authorization in many countries with hundreds of million of doses given worldwide (https://covid19.who.int).

While vaccines administered by IM injection induce robust systemic immunity that protects against severe disease and mortality, questions remain as to their ability to curtail SARS-CoV-2 transmission, especially if upper-airway infection is not reduced. Indeed, many of the IM-administered vaccines showed variable protection against upper-airway infection and transmission in pre-clinical studies and failed to induce substantive mucosal (immunoglobulin A [IgA]) immunity (Mercado et al., 2020; van Doremalen et al., 2020; Wang et al., 2020; Yu et al., 2020). This issue is important because of the emergence of more transmissible SARS-CoV-2 variants including B.1.1.7 (Alpha), B.1.351 (Beta), B.1.1.28/P.1 (Gamma), and B.1.617.2 (Delta) with substitutions in the spike protein. Experiments with pseudoviruses and authentic SARS-CoV-2 strains also suggest that neutralization by vaccine-induced sera is diminished against variants expressing mutations in the spike gene at positions L452, E484, and elsewhere (Chen et al., 2021; McCallum et al., 2021; Tada et al., 2021; Wang et al., 2021a, 2021b; Wibmer et al., 2021). Beyond possible negative impacts on protection, the combination of diminished immunity against certain variants and naturally lower anti-S IgG levels in the respiratory mucosa could create conditions for further selection of resistance in the upper airway and transmission into the general population.

We recently described a single-dose, intranasally (IN)-delivered chimpanzee Adenovirus (simian Ad-36)-based SARS-CoV-2 vaccine (ChAd-SARS-CoV-2-S) encoding a pre-fusion stabilized S protein that induced robust humoral, cell-mediated, and mucosal immune responses and limited upper- and lower-airway infection in K18-hACE2 transgenic mice, hamsters, and non-human primates (Bricker et al., 2021; Hassan et al., 2020b, 2021). This vaccine, which has advanced to human clinical trials (BBV154, Clinical Trial NCT04751682), differs from ChAdOx1 nCoV-19, a chimpanzee Ad-23-based SARS-CoV-2 vaccine, currently granted emergency use in some countries. Here, as a further step to evaluating the potential utility of ChAd-SARS-CoV-2-S, we assessed its dose response, durability, and cross-protective activity in mice including effects on upper- and lower-airway infection. At approximately 9 months after IN immunization, neutralizing antibody and anti-S protein IgG and IgA levels in serum of ChAd-SARS-CoV-2-S-vaccinated animals remained high and inhibited infection with SARS-CoV-2 strains with B.1.351, and B.1.1.28 spike proteins. At this time point, susceptible K18-hACE2 transgenic mice were fully protected against upper and lower respiratory tract infection after challenge with a SARS-CoV-2 virus displaying B.1.351 spike proteins.

RESULTS

A single ChAd-SARS-CoV-2-S immunization induces durable anti-spike and neutralizing responses at different doses

We assessed the durability of humoral immune responses in BALB/c mice 100 or 200 days post-IM or IN immunization with escalating doses of ChAd-SARS-CoV-2-S (10^8, 10^9, and 10^{10} viral particles [vp]) or 10^{10} vp of a ChAd-Control vaccine (Figure 1A). First, we measured anti-S and anti-RBD IgG and IgA levels by ELISA. Consistent with prior results at a 1-month time point (Hassan et al., 2020b), at 100 or 200 days post-vaccination, IN immunization with ChAd-SARS-CoV-2-S induced superior antibody responses in serum than IM immunization or vaccination with ChAd-Control (Figures 1B–1M and S1). At 100 days after IN immunization with 10^{10}, 10^9, and 10^8 vp of ChAd-SARS-CoV-2-S, geometric mean titers (GMTs) of serum S-specific IgG responses were 1.1×10^6, 4.8×10^5, and 2.6×10^5, and RBD-specific IgG were 3.2×10^5, 1.8×10^5, and 8.7×10^4, respectively (Figure 1B). In comparison, S- and RBD-specific IgG responses 100 days after IM immunization with 10^{10}, 10^9, and 10^8 vp of ChAd-SARS-CoV-2-S were 4- to 6-fold lower ($p < 0.0001$) with S-specific IgG titers of 2.1×10^5, 1.1×10^5, and 4.5×10^4 and RBD-specific IgG titers of 5.1×10^4, 2.9×10^4, and 2.3×10^4, respectively (Figure 1E). A similar dose response was observed with S-and RBD-specific IgG titers in serum at 200 days after IN or IM immunization (Figures 1H and1 K). At 200 days after IN immunization with 10^{10}, 10^9, and 10^8 vp of ChAd-SARS-CoV-2-S, GMT of S-specific IgG were 2.8×10^6, 2.4×10^6, and 1.2×10^6, and RBD-specific IgG were 1.1×10^6, 6.1×10^5, and 3.2×10^5, respectively (Figure 1H). At 200 days after IM immunization with 10^{10}, 10^9, and 10^8 vp of ChAd-SARS-CoV-2-S, S-specific IgG GMT were 8.1×10^5, 6.9×10^5, and 2.6×10^5, and RBD-specific IgG GMT were 1.4×10^5, 1.3×10^5, and 8.0×10^4, respectively

Figure 1. ChAd-SARS-CoV-2-S induces durable immunity

(A) Immunization scheme. Five-week old female BALB/c mice were vaccinated via IN or IM route with 10^{10} viral particles of ChAd control or decreasing doses (10^{10}, 10^9, and 10^8 vp) of ChAd-SARS-CoV-2-S.

(B–M) Humoral responses in sera of immunized mice were evaluated (n = 6–14). An ELISA measured anti-S and RBD IgG and IgA levels from IN-immunized mice at 100 (B and C) or 200 (H and I) days post-vaccination, or from IM-immunized mice at 100 (E and F) or 200 (K and L) days post-vaccination. Neutralizing activity of sera determined by FRNT from IN- (D and J) or IM- (G and M) immunized mice at 100 (D and G) or 200 (J and M) days post-vaccination.

(N) The frequency of S-specific IgG- or IgA-producing LLPCs in the bone marrow measured by ELISPOT assay (n = 4).

For (B)–(M), one-way ANOVA with a Dunnett's post-test comparing vaccine and control groups: ns, not significant; $^{**}p < 0.01$; $^{****}p < 0.0001$). For (N), Mann-Whitney test: $^{*}p < 0.05$. (B–N) Bars show median values, and dotted lines indicate the limit of detection (LOD) of the assays.

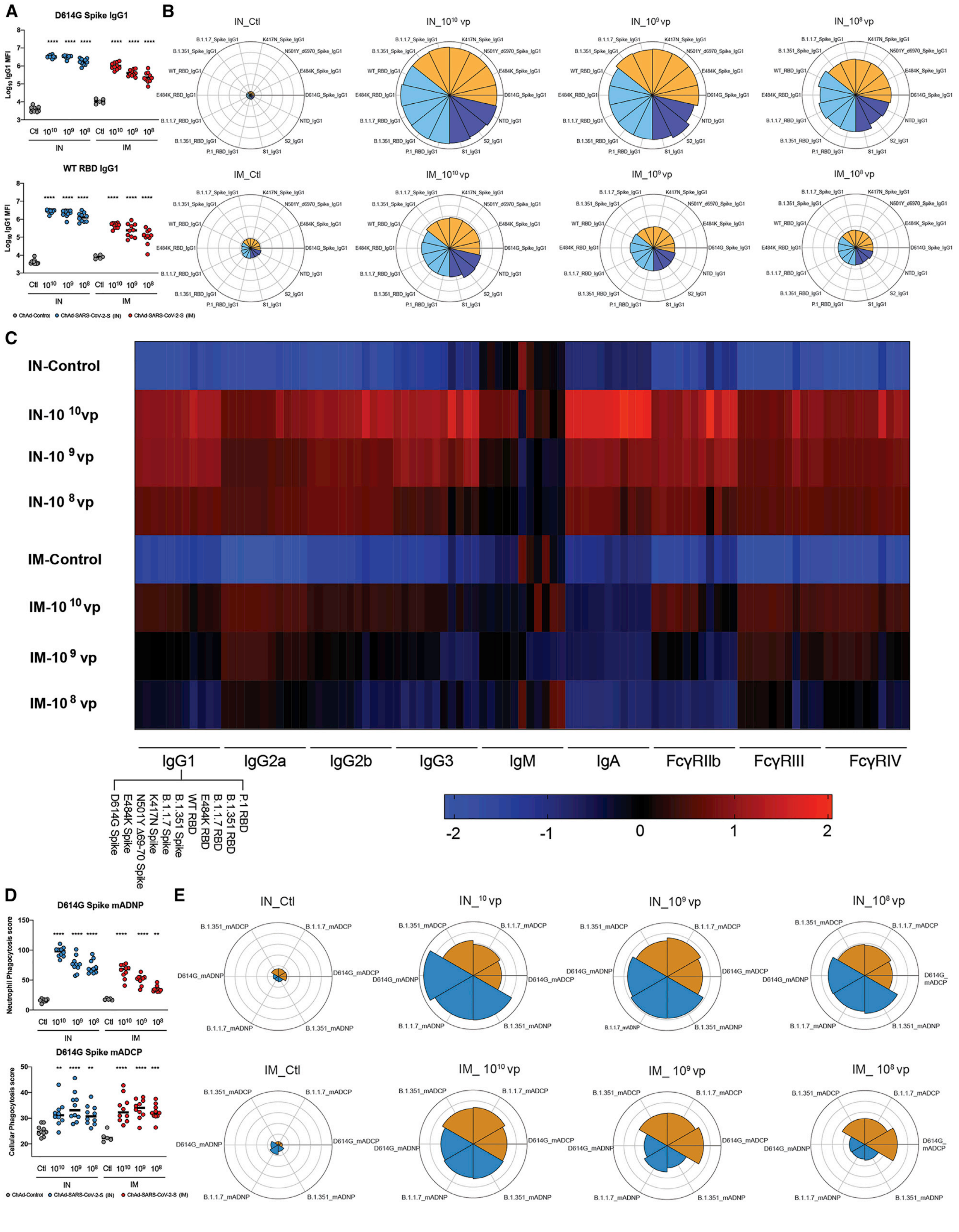

(legend on next page)

(Figure 1K). Thus, anti-S and anti-RBD IgG levels were higher after IN than IM immunization and continued to rise in serum even several months after single-dose vaccination.

We next assessed the induction and durability of serum IgA responses. Although IM immunization failed to induce S- or RBD-specific IgA (Figures 1F and 1L), substantial levels of anti S- and RBD IgA were detected after IN immunization at 100 or 200 days post-immunization (Figures 1C and 1I). At 100 days after IN immunization with 10^{10}, 10^9, and 10^8 vp of ChAd-SARS-CoV-2-S, the GMT of S-specific IgA were 4.8×10^3, 1.2×10^3, and 8.4×10^2, and RBD-specific IgA were 2.2×10^3, 4.6×10^2, and 2.9×10^2, respectively (Figure 1C). As seen with IgG, the IgA levels continued to increase over time such that at 200 days the GMT of S-specific IgA were 1.1×10^4, 7.4×10^3, and 5.4×10^3, and RBD-specific IgA were 5.2×10^3, 3.8×10^3, and 9.8×10^2 after IN immunization with 10^{10}, 10^9, and 10^8 vp of ChAd-SARS-CoV-2-S, respectively (Figure 1I).

We next evaluated a functional correlate of the serological response by assaying neutralizing activity (Figures 1D, 1G, 1J, 1M, and S1) using a focus-reduction neutralization test (FRNT) (Case et al., 2020). As expected, neutralizing activity was not detected in sera from ChAd-control-treated mice. At 100 days post-IN immunization with 10^{10}, 10^9, and 10^8 vp of ChAd-SARS-CoV-2-S, the mean effective half maximal inhibitory titers [EC_{50}] were 39,449, 9,989, and 7,270, respectively (Figure 1D). In comparison, at this time point after IM immunization with 10^{10}, 10^9, and 10^8 vp of ChAd-SARS-CoV-2-S, the EC_{50} values were 8- to 20-fold lower ($p < 0.0001$) at 4,988, 2,017, and 391, respectively (Figure 1G). At 200 days after IN immunization with 10^{10}, 10^9, and 10^8 vp of ChAd-SARS-CoV-2-S, and consistent with the higher anti-S and RBD titers seen, EC_{50} values were 45,591, 22,769, and 23,433, respectively (Figure 1J). In comparison, 200 days after IM immunization with 10^{10}, 10^9, and 10^8 vp of ChAd-SARS-CoV-2-S, EC_{50} values were much lower at 2,524, 940, and 716, respectively (Figure 1M).

Long-lived plasma cells (LLPCs) reside in the bone marrow and constitutively secrete high levels of antibody that correlate with serum levels (Amanna and Slifka, 2010). To assess the levels of antigen-specific LLPCs at 200 days after IM or IN immunization with 10^{10} vp of ChAd-SARS-CoV-2-S, $CD138^+$ cells were isolated from the bone marrow and assayed for S-specific IgG or IgA production using an ELISPOT assay (Purtha et al., 2011). We observed an ~4-fold higher frequency of LLPCs secreting S-specific IgG after IN immunization than IM immunization (Figure 1N). Additionally, after IN immunization, we detected LLPCs producing S-specific IgA, which were absent after IM immunization (Figure 1N). Together, these data establish the following: (1) single-dose IN immunization promotes superior humoral immunity than IM immunization, (2) 100-fold lower inoculating doses of ChAd-SARS-CoV-2-S induce robust neutralizing antibody responses in mice, (3) IN but not IM immunization induces serum IgA responses and IgA-specific LLPCs against the SARS-CoV-2 S protein, and (4) the humoral immunity induced by ChAd-SARS-CoV-2-S is durable and rises over a 6-month period after vaccination.

IN inoculation of ChAd-SARS-CoV-2-S induces broad antibody responses with Fc effector function capacity

To characterize the humoral response further, we analyzed antibody binding to SARS-CoV-2 variant proteins and Fc effector functions using serum derived from BALB/c mice at 90 days after IN or IM vaccination. Our panel of SARS-CoV-2 proteins included spike (D614G, E484K, N501Y, Δ69-70, K417N) and RBD (E484K) antigens corresponding to WA1/2020, B.1.1.7, B.1.351, and B.1.1.28 strains. We first measured the anti-SARS-CoV-2 specific antibody response for several isotypes (IgG1, IgG2a, IgG2b, IgG3, IgM, and IgA) and their ability to bind Fcγ receptors (mouse FcγRIIB, FcγRIII, and FcγRIV) using a luminex platform. Consistent with data obtained by ELISA (Figures 1B and 1E), IN vaccination of ChAd-SARS-CoV-2-S induced higher levels of IgG1 to D614G spike and WA1/2020 RBD proteins than IM immunization, and as expected, decreasing doses of the vaccine elicited lower antibody titers (Figure 2A). Anti-SARS-CoV-2 IgG1 titers after IN immunization also were higher against all spike and RBD variants than after IM immunization, and titers decreased with vaccine dose (Figure 2B). As shown in a heatmap, this trend was observed for all anti-SARS-CoV-2 specific antibody isotypes and correlated with FcγR binding patterns (Figure 2C). These data suggest that IN vaccination induces a higher magnitude and broader antibody subclass response to SARS-CoV-2 than IM vaccination.

Antibody effector functions, such as opsonization, are mediated in part by Fcγ receptor engagement (Bruhns and Jönsson, 2015). To determine whether the observed differences in antibody titers and FcγR binding titers resulted in differences in effector functions, we performed antibody-dependent neutrophil (ADNP) and cellular phagocytosis (ADCP) assays (Figures 2D and 2E). Sera from IN-vaccinated mice stimulated substantially more ADNP than those obtained from IM-vaccinated mice. However, minimal differences in ADCP were apparent from

Figure 2. Intranasal inoculation of ChAd-SARS-CoV-2-S induces antibody responses with Fc effector function capacity

(A) Serum was analyzed by Luminex platform to quantify the amount of anti-SARS-CoV-2 (WA1/2020 D614G) spike and RBD IgG1. Bars represent the mean values.

(B) Serum was analyzed by Luminex to quantify the amount of anti-SARS-CoV-2 IgG1 to different SARS-CoV-2 protein variants. Polar plots represent the IgG1 median percentile rank for each SARS-CoV-2 protein and variant.

(C) Heatmap shows the IgG titer and FcγR binding titer of each vaccine regimen to SARS-CoV-2 Spike or RBD proteins. Each square represents the average *Z* score within a group for the condition.

(D) Serum was incubated with primary mouse neutrophils (mADNP) or J774A.1 cells (mADCP) and SARS-CoV-2 spike-coated beads, and phagocytosis was measured after 1 h. Bars represent the mean and the error bars indicate standard deviations.

(E) Serum was incubated with primary mouse neutrophils (mADNP) or J774A.1 cells (mADCP) and WA1/2020 D614G, B.1.1.7, or B1.351 spike-coated beads, and phagocytosis was measured after 1 h. Polar plots represent the mADNP or mADCP median percentile rank for each SARS-CoV-2 protein and variant.

For (A and D), one-way ANOVA with a Dunnett's post-test comparing vaccine to control groups: **$p < 0.01$; ***$p < 0.001$; ****$p < 0.0001$). In (A) and (D), bars indicate median values.

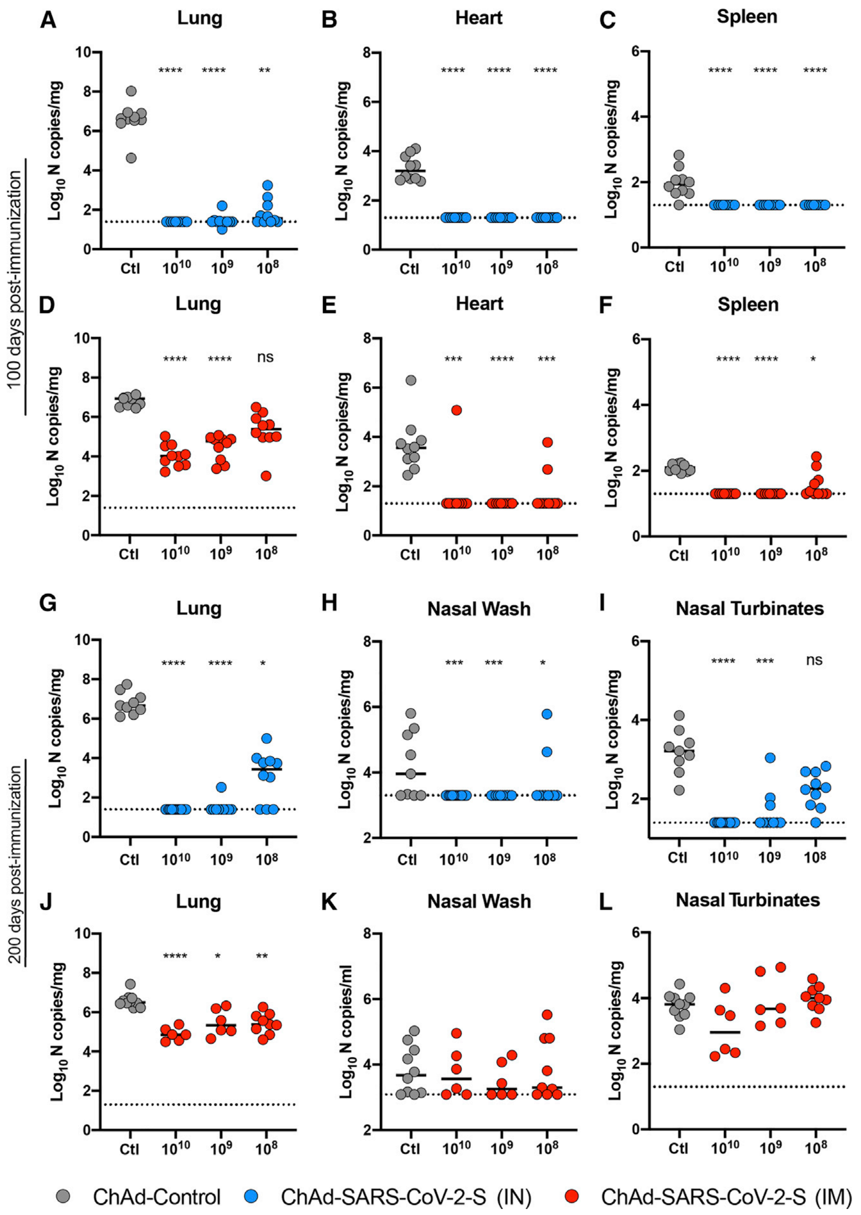

Figure 3. Durability of protective efficacy of ChAd-SARS-CoV-2-S against SARS-CoV-2 infection in BALB/c mice

(A–L) Five-week old female BALB/c mice were immunized via IN or IM route with 10^{10} vp of ChAd control or 10^{10}, 10^9, and 10^8 vp of ChAd-SARS-CoV-2-S. On day 100 or 200 post-immunization, mice were challenged as follows: animals were treated with anti-Ifnar1 mAb and transduced with Hu-AdV5-hACE2 via an IN route 1 day later. Five days later, mice were inoculated with 5 × 10^4 FFU of SARS-CoV-2 WA1/2020 via the intranasal route. Tissues were harvested at 4 dpi, and viral RNA levels were measured from mice challenged 100 (A–F) or 200 (G–L) days post-immunization by qRT-PCR (n = 6–14, Kruskal Wallis with Dunn's post-test: ns, not significant; **$p < 0.01$; *$p < 0.1$; ***$p < 0.001$ ****$p < 0.0001$). Bars show median values, and dotted lines indicate the LOD of the assays.

antibodies derived after IN and IM vaccination (Figures 2D and 2E). These data demonstrate that IN vaccination with ChAd-SARS-CoV-2-S induces a greater and more functional antibody response than after IM vaccination.

Intranasally administered ChAd-SARS-CoV-2-S induces durable protection against SARS-CoV-2 challenge in BALB/c mice

To assess the efficacy of the ChAd-SARS-CoV-2-S vaccine, immunized BALB/c mice given the dosing regimen described in Figure 1A were challenged with SARS-CoV-2. Virus challenge was preceded by intranasal introduction of Hu-Ad5-hACE2, which enables ectopic expression of hACE2 and productive infection of SARS-CoV-2 in BALB/c mice by historical SARS-CoV-2 strains (Hassan et al., 2020a; Sun et al., 2020). Animals were immunized once via IN or IM routes with 10^{10} vp of ChAd-Control or 10^8, 10^9, or 10^{10} vp of ChAd-SARS-CoV-2-S. At day 95 or 195 post-vaccination, mice were given 10^8 plaque-forming units (PFUs) of Hu-Ad5-hACE2 and anti-Ifnar1 mAb; the latter attenuates innate immunity and enhances pathogenesis in this model (Hassan et al., 2020a). Five days later, BALB/c mice were challenged with 5 × 10^4 focus-forming units (FFUs) of SARS-CoV-2 (strain WA1/2020) via IN route. At 4 days post-infection (dpi), lungs, spleen, and heart were harvested from mice challenged at 100 days post-immunization, and lungs, nasal turbinates, and nasal washes were collected from a second cohort challenged at 200 days post-immunization. Tissues were assessed for viral burden by quantitative reverse transcription PCR (qRT–PCR) using primers for the subgenomic RNA (N gene). IN immunization with all three doses induced remarkable protection at 100 days post-vaccination as evidenced by a virtual absence of viral RNA in lungs, spleen, and heart compared to animals receiving the ChAd-Control vaccine (Figures 3A–3C). At 200 days post-immunization, protection conferred by the IN delivered ChAd-SARS-CoV-2-S remained robust in the upper and lower respiratory tracts compared to ChAd-Control immunized mice. Nevertheless, we observed limited infection breakthrough in the lungs and nasal turbinates in animals immunized with the lowest 10^8 vp dose of ChAd-SARS-CoV-2-S (Figures 3G and 3I). In comparison, protection at 100 days post-IM immunization was less than after IN immunization at the same challenge time point. Although viral RNA was not detected in the heart and spleen (Figure 3E-F), at least 1,000- to 30,000-fold ($p < 0.0001$) higher levels were measured in the lungs of mice immunized with ChAd-SARS-CoV-2-S by

CellPress
OPEN ACCESS

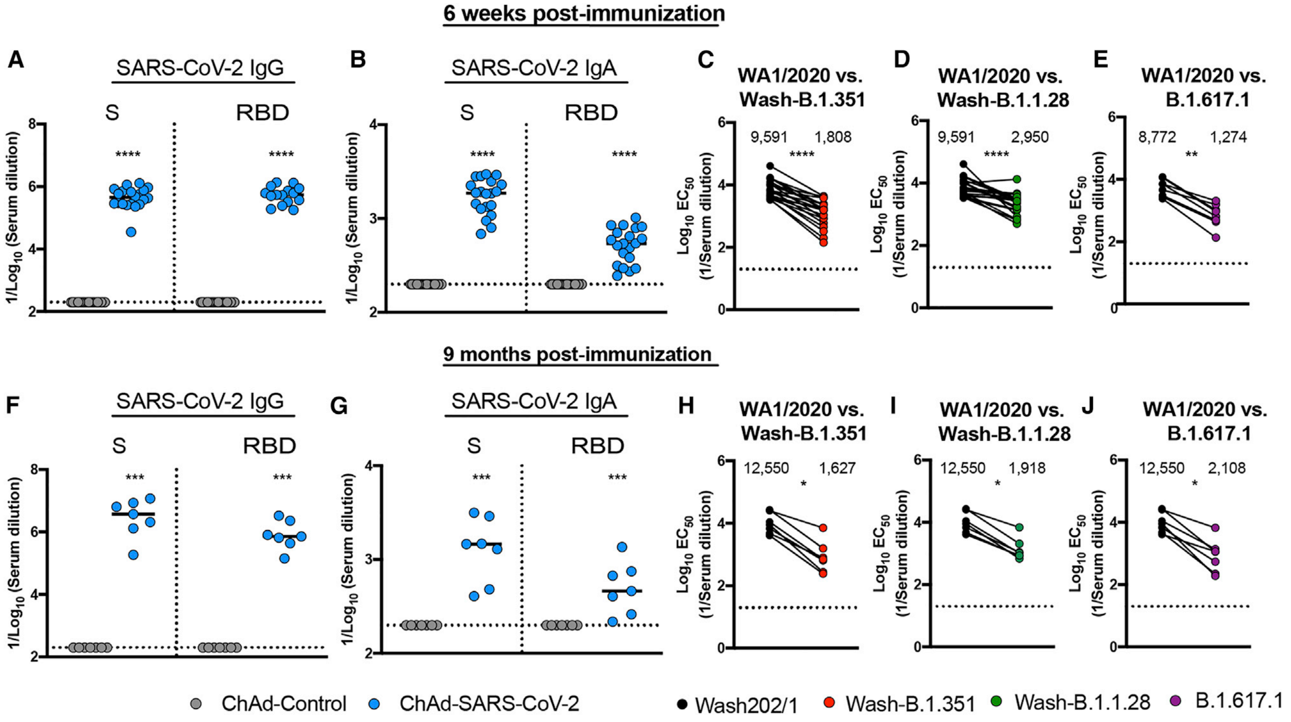

Figure 4. Immunogenicity of intranasal administration ChAd-SARS-CoV-2-S in K18-hACE2 mice

(A–J) Five-week-old K18-hACE2 female mice were immunized with ChAd control or ChAd-SARS-CoV-2-S via an IN route. Antibody responses in sera of mice at 6 weeks (A–E, n = 20) or 9 months (F–J, n = 7) after immunization were evaluated. An ELISA measured SARS-CoV-2 S- and RBD-specific IgG (A, F) and IgA levels (B and G), and an FRNT determined neutralization activity (C–E and H–J). Paired analysis of serum neutralizing activity from immunized mice collected at 6 weeks (C–E) or 9 months (H–J) against WA1/2020 and Wash-B.1.351 (C and H), Wash-B.1.1.28 (D and I), or B.1.617.1 (E and J). (A, B, F, and G) Mann-Whitney test: ***$p < 0.001$; ****$p < 0.0001$. (C–E and H–J) Two-tailed Wilcoxon matched-pairs signed rank test: *$p < 0.05$; **$p < 0.01$; ****$p < 0.0001$.

the IM compared to IN route (Figures 3A and 3D). We also observed a greater impact of dosing by the IM route, as the reduction in viral RNA load in the lungs at 10^8 vp dose no longer was different than in the ChAd-control-vaccinated mice (Figure 3D). At 200 days post-IM immunization, we observed less protection against SARS-CoV-2 infection in the lungs, nasal washes, and nasal turbinates than after IN immunization (Figures 3G–3L).

ChAd-SARS-CoV-2-S induces durable immunity in hACE2 transgenic mice

We next assessed the immunogenicity of intransally delivered ChAd-SARS-CoV-2-S in K18-hACE2 C57BL/6 mice, which are more vulnerable to SARS-CoV-2 infection than BALB/c mice (Golden et al., 2020; Winkler et al., 2020; Yinda et al., 2021). Five-week old K18-hACE2 mice were inoculated via an IN route with 10^9 vp of ChAd control or ChAd-SARS-CoV-2-S. Serum samples were collected 6 weeks later, and humoral immune responses were evaluated. IN immunization of ChAd-SARS-CoV-2-S but not ChAd control induced high levels of S- and RBD-specific IgG and IgA (Figures 4A and 4B). Neutralizing antibody titers against WA1/2020 and three other SARS-CoV-2 strains with spike proteins from B.1.351 and B.1.1.28 variants or a B.1.617.1 isolate were measured by FRNT assay (Figures 4C–4E and S2). High levels of neutralizing antibody against WA1/2020 were induced after a single IN dose of ChAd-SARS-CoV-2-S. As seen with vaccine-induced human sera against some variant viruses (Chen et al., 2021; McCallum et al., 2021; Tada et al., 2021; Wang et al., 2021a, 2021b; Wibmer et al., 2021), we observed decreases in neutralizing titers against Wash-B.1.351 (~5-fold, $p < 0.0001$; Figure 4C), Wash-B.1.1.28 (~3-fold, $p < 0.0001$; Figure 4D), and B.1.617.1 (~7-fold, $p < 0.01$, Figure 4E) strains compared to WA1/2020. To assess the durability of humoral responses, a separate cohort of K18-hACE2 mice was immunized via the IN route, and serum samples were collected at 9 months. ChAd-SARS-CoV-2-S induced high levels of S- and RBD-specific IgG and IgA and neutralizing antibody against WA1/2020 (EC_{50} of 12,550) at this time point (Figures 4F–4J and S2). When tested against the Wash-B.1.351, Wash-B.1.1.28, and B.1.617.1 viruses, we also observed a diminished neutralizing titer (~6- to 8-fold, $p < 0.05$; Figures 4H–4J) compared to WA1/2020, although they still remained high (EC_{50} of 1,627, 1,918, and 2,108, respectively).

ChAd-SARS-CoV-2-S confers cross-protection against Wash B.1.351 and Wash-B.1.1.28 challenge in hACE2 transgenic mice

We tested the protective efficacy of ChAd-SARS-CoV-2-S against WA1/2020 and two chimeric viruses (Wash-B.1.351 and Wash-B.1.1.28) with spike genes corresponding to variants

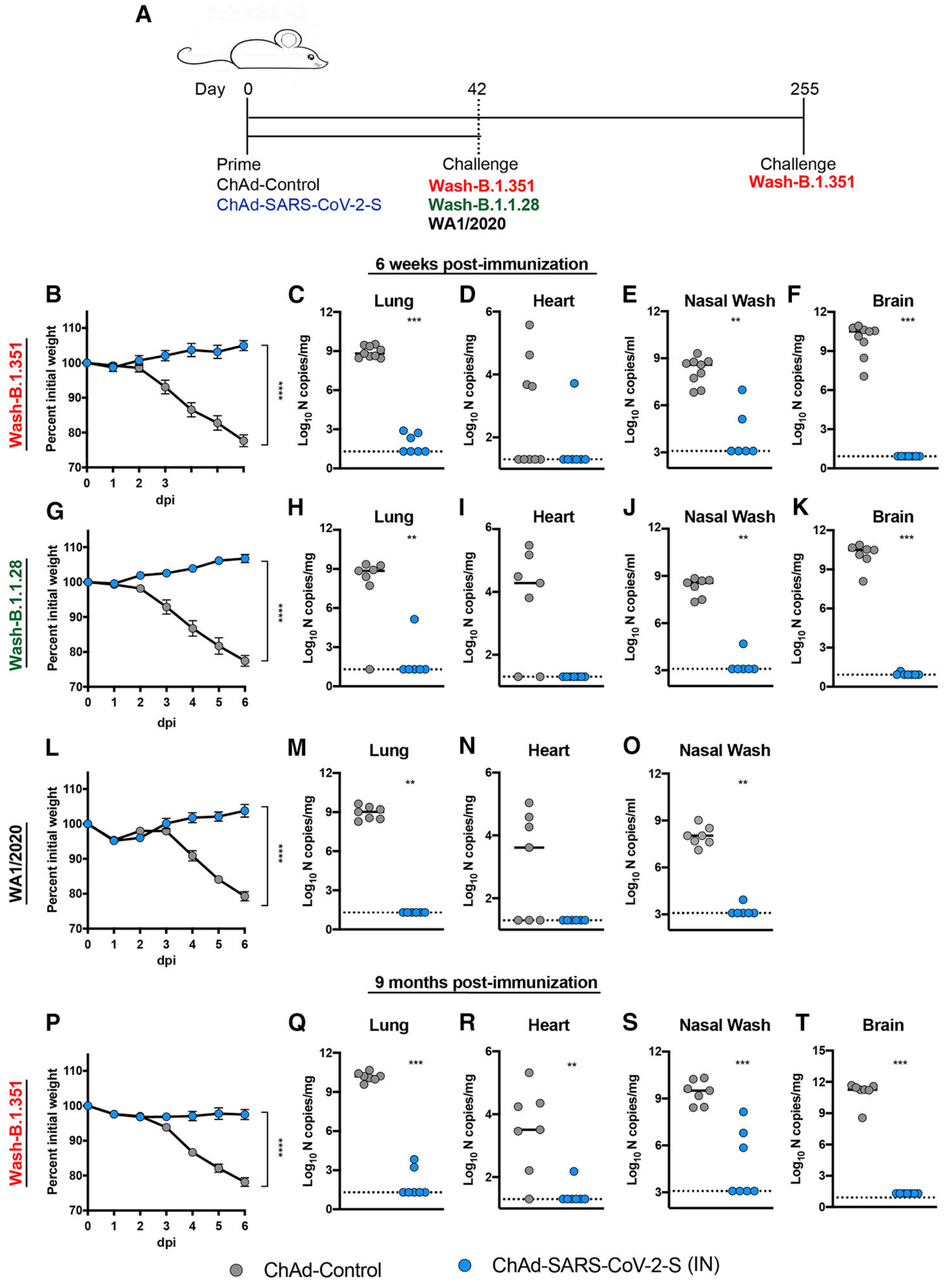

Figure 5. ChAd-SARS-CoV-2-S confers cross-protection against variant viruses in K18-hACE2 mice

(A) Experimental scheme. Five-week-old K18-hACE2 female mice were immunized via an IN route with ChAd control or ChAd-SARS-CoV-2-S.

(B–O) At 6 weeks post-immunization, mice were challenged with 10^4 FFU of SARS-CoV-2 of Wash-B.1.351 (B–F), Wash-B.1.1.28 (G–K), or WA1/2020 (L–O).

(P–T) At 9 months post-immunization, mice were challenged with 10^4 FFU of Wash-B.1.351.

(B, G, L, and P) Body-weight change over time. Data are the mean ± SEM comparing vaccine to control groups (n = 6–9 for each group; unpaired t test for area under curve, ****$p < 0.0001$).

(C–F, H–K, M–O, and Q–T) Viral RNA levels in the lung, heart, brain, and nasal washes were measured at 6 dpi by qRT-PCR (n = 6–9; Mann-Whitney test: **$p < 0.01$, ***$p < 0.001$). Bars show median values, and dotted lines indicate the LOD of the assays.

of concern (Figure 5A). Five-week old K18-hACE2 mice were immunized via an IN route with a single 10^9 vp dose of ChAd control or ChAd-SARS-CoV-2-S. Six weeks later, mice were challenged by an IN route with 10^4 FFU of Wash-B.1.351, Wash B.1.1.28, or WA1/2020. All mice immunized with ChAd-SARS-CoV-2 exhibited no weight loss, whereas most ChAd-Control-vaccinated mice experienced substantial weight loss at 3 to 6 dpi (Figures 5B, 5G, and 5L). Remarkably, vaccination with ChAd-SARS-CoV-2-S resulted in almost no detectable SARS-CoV-2 RNA in the upper and lower respiratory tracts, heart,

and brain at 6 dpi (Figures 5C–5F, 5H–5K, and 5M–5O). As a further test of the durability of the cross-protective response, 5-week-old K18-hACE2 mice were immunized via an IN route with a single 10^{10} vp dose of ChAd control or ChAd-SARS-CoV-2-S. Nine months later, mice were challenged via IN route with 10^4 FFU of Wash-B.1.351. ChAd-SARS-CoV-2-S-vaccinated mice maintained weight in contrast to ChAd-Control-treated mice (Figure 5P). Moreover, substantial virological protection was observed, as only very low amounts of Wash-B.1.351 SARS-CoV-2 RNA were detected in the upper and lower respiratory tracts, heart, and brain in some of the mice (Figures 5Q–5T).

DISCUSSION

The durability of vaccine-induced immune responses is a key for providing sustained protection against SARS-CoV-2 infection and curtailing the current pandemic. Here, we show that a single IN immunization with ChAd-SARS-CoV-2-S induced S- and RBD-specific binding and neutralizing antibodies that continued to rise for several months, suggestive of sustained germinal center reactions. LLPCs in the bone marrow were detected 6 months after IN vaccination, secreting SARS-CoV-2-specifc IgG and IgA that likely contributed to the durably high antiviral antibody levels in circulation (Amanna and Slifka, 2010). In comparison, IM immunization with ChAd-SARS-CoV-2-S induced lower levels of serum neutralizing antibodies, fewer spike-specific IgG secreting LLPCs, and virtually no serum or cellular IgA response. At least in mice, a single IN dose immunization with ChAd-SARS-CoV-2-S produced durable humoral immunity that was observed across a 100-fold dose range. These pre-clinical immunogenicity results compare favorably with studies in humans with mRNA vaccines against SARS-CoV-2, which show humoral immune responses lasting at least several months (Doria-Rose et al., 2021; Widge et al., 2021). In comparison, the durability of antibody responses after natural SARS-CoV-2 infection can vary considerably (Dan et al., 2021; Gudbjartsson et al., 2020).

A single immunization of ChAd-SARS-CoV-2-S conferred durable protection against SARS-CoV-2 (WA1/2020 strain) challenge in hACE2-tranduced BALB/c mice or K18-hACE2 transgenic C57BL/6 mice at multiple time points. IN immunization in particular provided virtually complete virological protection against upper and lower respiratory tract infection, with only a limited infection breakthrough seen at the 100-fold lower vaccine dose. The abrogation of infection in the upper respiratory tract suggests that IN vaccination could prevent transmission, although corroborating studies are needed in other rodent (e.g., hamsters or ferret) models better suited to studying this question (Muñoz-Fontela et al., 2020). In comparison, IM immunization reduced the viral RNA levels in the lungs but showed substantially less protection against the homologous WA1/2020 strain in samples from the upper respiratory tract. While many SARS-CoV-2 vaccine candidates from different platforms have demonstrated immunogenicity and protective efficacy in animals models (García-Arriaza et al., 2021; Hennrich et al., 2021; Tostanoski et al., 2020; van Doremalen et al., 2020; Vogel et al., 2021; Yao et al., 2021; Yu et al., 2020), to our knowledge, none have established durability or protection against variant viruses. The long-term protection conferred by IN immunization even at 100-fold lower inoculating doses is promising but remains to be validated in human clinical trials with ChAd-SARS-CoV-2-S. If results in mice were recapitulated, dose sparing strategies could enable production of a large number of vaccine doses that could curtail infection and transmission of SARS-CoV-2.

The emergence of SARS-CoV-2 S variants with mutations of amino acids in the receptor binding motif (e.g., B.1.351, B.1.1.28, and B.1.617) is of concern because of their resistance to the inhibitory activity of many neutralizing antibodies (Chen et al., 2021; Wang et al., 2021a, 2021b). Indeed, human sera from subjects vaccinated with BNT162b2 mRNA or ChAdOx1 nCoV-19 (AZD1222) vaccines showed reduced neutralization against B.1.351 (Chen et al., 2021; Madhi et al., 2021; Zhou et al., 2021). Concerningly, IM-administered ChAdOx1 nCoV-19 (AZD1222) showed reduced protective efficacy against mild to moderate B.1.351 infection in humans (Madhi et al., 2021). In K18-hACE2 transgenic mice, when we compared the immunogenicity of IN-delivered ChAd-SARS-CoV-2-S against WA1/2020 and chimeric SARS-CoV-2 strains expressing B.1.1.28 or B.1.351 spike proteins or a B.1.617.1 isolate, we also observed reduced (3- to 8-fold) neutralization of the variant viruses although the titers remained >1,000. At 6 weeks post-IN immunization of ChAd-SARS-CoV-2-S, K18-hACE2 mice were fully protected against weight loss and infection in the upper and lower respiratory tracts and brain by WA1/2020, Wash-B.1.351, and Wash-B.1.1.28. Remarkably, in a separate cohort of K18-hACE2 mice challenged 9 months after single IN immunization, animals maintained high neutralizing titers against all of the variant strains and were fully protected against Wash-B.1.351 challenge. Although correlates of protection are not fully established for SARS-CoV-2 vaccines, the high levels of cross-neutralizing antibodies against the variant viruses combined with robust virus-specific systemic and mucosal $CD8^+$ T cell responses described previously (Hassan et al., 2020b) likely contribute to protection. Beyond this, antibody effector functions also might contribute to prevent SARS-CoV-2 infection and disease (Bartsch et al., 2021; Schäfer et al., 2021; Winkler et al., 2021). Indeed, we observed enhanced Fc effector functions against SARS-CoV-2 variant proteins in serum derived from IN-delivered ChAd-SARS-CoV-2-S including robust induction of ADNP and ADCP responses.

Limitations of study

Although a single intranasal administration of ChAd-SARS-CoV-2-S durably protected against SARS-CoV-2 variant replication in the upper and lower respiratory tracts even ~9 months after immunization, we note several limitations in our study. (1) We performed challenge studies in BALB/c mice transduced with hACE2 or C57BL/6 mice expressing an hACE2 transgene. Durability and protection studies will need to be corroborated in hamsters, non-human primates, and ultimately in humans. (2) Although our studies suggest that the mucosal immunity induced by intranasal vaccination could limit SARS-CoV-2 transmission, the use of mice precluded formal respiratory transmission analysis, which is better studied in hamsters and ferrets (Muñoz-

Fontela et al., 2020). (3) We observed robust protection *in vivo* against viruses displaying B.1.351 and B.1.1.28 spike proteins likely due to the high serum neutralizing antibody titers. Even though neutralizing antibody levels were lower with the variant strains due to mutations at sites in the receptor binding motif, the high starting level against the historical SARS-CoV-2 likely provided a sufficient cushion to overcome this loss in potency. Studies in other animals or with even lower doses of vaccine where neutralizing titers might be lower are needed to determine whether the protective phenotype against variants of concern is maintained. (4) Finally, we did not establish the correlates of protection in these studies, as passive antibody transfer or T cell depletions were not performed. Such studies could be performed in follow-up experiments.

In summary, our study shows that IN immunization with ChAd-SARS-CoV-2-S induces robust and durable binding IgG and IgA antibody, neutralizing antibody, Fc effector functions, and LLPC responses against SARS-CoV-2. In mice, a single IN immunization with ChAd-SARS-CoV-2-S confers cross-protection against SARS-CoV-2 strains displaying spike proteins corresponding to B.1.351, B.1.1.28, and B.1.617.1 variants, even 9 months after vaccination. Given the efficacy of preclinical evaluation in multiple animal models (Bricker et al., 2021; Hassan et al., 2021; Hassan et al., 2020b) and the durable protective immunity against variants of concern, IN delivery of ChAd-SARS-CoV-2-S may be a promising platform for preventing SARS-CoV-2 infection, curtailing transmission, and, thus, warrants further clinical evaluation in humans.

STAR★METHODS

Detailed methods are provided in the online version of this paper and include the following:

- KEY RESOURCES TABLE
- RESOURCE AVAILABILITY
  - Lead contact
  - Materials availability
  - Data and code availability
- EXPERIMENTAL MODEL AND SUBJECT DETAILS
  - Viruses and cells
  - Mouse experiments
- METHOD DETAILS
  - Chimpanzee and human adenovirus vectors
  - SARS-CoV-2 neutralization assays
  - Protein expression and purification
  - ELISA
  - ELISPOT assay
  - Measurement of viral burden
  - Luminex analysis
  - Antibody-dependent neutrophil or cellular phagocytosis
- QUANTIFICATION AND STATISTICAL ANALYSIS

SUPPLEMENTAL INFORMATION

Supplemental information can be found online at https://doi.org/10.1016/j.celrep.2021.109452.

ACKNOWLEDGMENTS

This study was supported by NIH contracts and grants (R01 AI157155, R01 EB026468-02S1, 75N93019C00062, 75N93021C00017, HHSN27220140001 8C, and U19 142790) and INV-00613 from the Bill and Melinda Gates Foundation. This work also was supported by Woodruff Health Sciences Center 2020 COVID-19 CURE Award.

AUTHOR CONTRIBUTIONS

A.O.H., I.P.D., and E.K. generated the vaccines. A.O.H. performed ELISA assays and analyzed the data. A.O.H. performed neutralization assays. S.R. performed ELISPOT assays. M.J.G., D.Y., and G.A. designed and/or performed the binding and Fc effector function experiments. L.J.A., D.H.F., C.M., and E.O.S. designed and produced the recombinant S and RBD proteins and antibodies. A.O.H., S.S., and B.Y. performed and evaluated the virological assays. P.-Y.S., M.E.D.-G., and M.S.S. provided key reagents. M.S.D., D.H.F., G.A., and D.T.C. designed experiments and secured funding. A.O.H., M.J.G., and M.S.D. wrote the initial draft, with the other authors providing editorial comments.

DECLARATION OF INTERESTS

M.S.D. is a consultant for Inbios, Vir Biotechnology, Fotress Biotech, and Carnival Corporation and on the Scientific Advisory Board of Moderna and Immunome. The Diamond laboratory has received unrelated funding support in sponsored research agreements from Moderna, Vir Biotechnology, and Emergent BioSolutions. M.S.D. is on the Advisory Board of Moderna. M.S.D., I.P.D., D.T.C., and A.O.H. have filed a disclosure with Washington University for possible commercial development of ChAd-SARS-CoV-2-S. D.T.C. is an equity holder in Precision Virologics, Inc., which has optioned the ChAd-SARS-CoV-2-S vaccine.

Received: May 13, 2021
Revised: June 11, 2021
Accepted: July 2, 2021
Published: July 27, 2021

REFERENCES

Alsoussi, W.B., Turner, J.S., Case, J.B., Zhao, H., Schmitz, A.J., Zhou, J.Q., Chen, R.E., Lei, T., Rizk, A.A., McIntire, K.M., et al. (2020). A Potently Neutralizing Antibody Protects Mice against SARS-CoV-2 Infection. J. Immunol. *205*, 915–922.

Amanna, I.J., and Slifka, M.K. (2010). Mechanisms that determine plasma cell lifespan and the duration of humoral immunity. Immunol. Rev. *236*, 125–138.

Baden, L.R., El Sahly, H.M., Essink, B., Kotloff, K., Frey, S., Novak, R., Diemert, D., Spector, S.A., Rouphael, N., Creech, C.B., et al.; COVE Study Group (2021). Efficacy and Safety of the mRNA-1273 SARS-CoV-2 Vaccine. N. Engl. J. Med. *384*, 403–416.

Barnes, C.O., Jette, C.A., Abernathy, M.E., Dam, K.A., Esswein, S.R., Gristick, H.B., Malyutin, A.G., Sharaf, N.G., Huey-Tubman, K.E., Lee, Y.E., et al. (2020). SARS-CoV-2 neutralizing antibody structures inform therapeutic strategies. Nature *588*, 682–687.

Barrett, J.R., Belij-Rammerstorfer, S., Dold, C., Ewer, K.J., Folegatti, P.M., Gilbride, C., Halkerston, R., Hill, J., Jenkin, D., Stockdale, L., et al.; Oxford COVID Vaccine Trial Group (2021). Phase 1/2 trial of SARS-CoV-2 vaccine ChAdOx1 nCoV-19 with a booster dose induces multifunctional antibody responses. Nat. Med. *27*, 279–288.

Bartsch, Y.C., Fischinger, S., Siddiqui, S.M., Chen, Z., Yu, J., Gebre, M., Atyeo, C., Gorman, M.J., Zhu, A.L., Kang, J., et al. (2021). Discrete SARS-CoV-2 antibody titers track with functional humoral stability. Nat. Commun. *12*, 1018.

Bricker, T.L., Darling, T.L., Hassan, A.O., Harastani, H.H., Soung, A., Jiang, X., Dai, Y.N., Zhao, H., Adams, L.J., Holtzman, M.J., et al. (2021). A single intranasal or intramuscular immunization with chimpanzee adenovirus vectored

SARS-CoV-2 vaccine protects against pneumonia in hamsters. Cell Reports *36*, 109400. https://doi.org/10.1016/j.celrep.2021.109400.

Brown, E.P., Dowell, K.G., Boesch, A.W., Normandin, E., Mahan, A.E., Chu, T., Barouch, D.H., Bailey-Kellogg, C., Alter, G., and Ackerman, M.E. (2017). Multiplexed Fc array for evaluation of antigen-specific antibody effector profiles. J. Immunol. Methods *443*, 33–44.

Bruhns, P., and Jönsson, F. (2015). Mouse and human FcR effector functions. Immunol. Rev. *268*, 25–51.

Burton, D.R., and Walker, L.M. (2020). Rational Vaccine Design in the Time of COVID-19. Cell Host Microbe *27*, 695–698.

Butler, A.L., Fallon, J.K., and Alter, G. (2019). A Sample-Sparing Multiplexed ADCP Assay. Front. Immunol. *10*, 1851.

Cao, L., Goreshnik, I., Coventry, B., Case, J.B., Miller, L., Kozodoy, L., Chen, R.E., Carter, L., Walls, A.C., Park, Y.J., et al. (2020a). De novo design of picomolar SARS-CoV-2 miniprotein inhibitors. Science *370*, 426–431.

Cao, Y., Su, B., Guo, X., Sun, W., Deng, Y., Bao, L., Zhu, Q., Zhang, X., Zheng, Y., Geng, C., et al. (2020b). Potent neutralizing antibodies against SARS-CoV-2 identified by high-throughput single-cell sequencing of convalescent patients' B cells. Cell *182*, 73–84.e16.

Case, J.B., Rothlauf, P.W., Chen, R.E., Liu, Z., Zhao, H., Kim, A.S., Bloyet, L.M., Zeng, Q., Tahan, S., Droit, L., et al. (2020). Neutralizing antibody and soluble ACE2 inhibition of a replication-competent VSV-SARS-CoV-2 and a clinical isolate of SARS-CoV-2. Cell Host Microbe *28*, 475–485.e5.

Chen, R.E., Zhang, X., Case, J.B., Winkler, E.S., Liu, Y., VanBlargan, L.A., Liu, J., Errico, J.M., Xie, X., Suryadevara, N., et al. (2021). Resistance of SARS-CoV-2 variants to neutralization by monoclonal and serum-derived polyclonal antibodies. Nat. Med. *27*, 717–726.

Cheung, E.W., Zachariah, P., Gorelik, M., Boneparth, A., Kernie, S.G., Orange, J.S., and Milner, J.D. (2020). Multisystem Inflammatory Syndrome Related to COVID-19 in Previously Healthy Children and Adolescents in New York City. JAMA *324*, 294–296.

Dan, J.M., Mateus, J., Kato, Y., Hastie, K.M., Yu, E.D., Faliti, C.E., Grifoni, A., Ramirez, S.I., Haupt, S., Frazier, A., et al. (2021). Immunological memory to SARS-CoV-2 assessed for up to 8 months after infection. Science *371*, eabf4063.

Doria-Rose, N., Suthar, M.S., Makowski, M., O'Connell, S., McDermott, A.B., Flach, B., Ledgerwood, J.E., Mascola, J.R., Graham, B.S., Lin, B.C., et al. (2021). Antibody Persistence through 6 Months after the Second Dose of mRNA-1273 Vaccine for Covid-19. N. Engl. J. Med. *384*, Published online June 10, 2021.

García-Arriaza, J., Garaigorta, U., Pérez, P., Lázaro-Frías, A., Zamora, C., Gastaminza, P., Del Fresno, C., Casasnovas, J.M., Sorzano, C.Ó.S., Sancho, D., and Esteban, M. (2021). COVID-19 vaccine candidates based on modified vaccinia virus Ankara expressing the SARS-CoV-2 spike induce robust T- and B-cell immune responses and full efficacy in mice. J. Virol. *95*, e02260-20.

Golden, J.W., Cline, C.R., Zeng, X., Garrison, A.R., Carey, B.D., Mucker, E.M., White, L.E., Shamblin, J.D., Brocato, R.L., Liu, J., et al. (2020). Human angiotensin-converting enzyme 2 transgenic mice infected with SARS-CoV-2 develop severe and fatal respiratory disease. JCI Insight *5*, e142032.

Graham, B.S. (2020). Rapid COVID-19 vaccine development. Science *368*, 945–946.

Gudbjartsson, D.F., Norddahl, G.L., Melsted, P., Gunnarsdottir, K., Holm, H., Eythorsson, E., Arnthorsson, A.O., Helgason, D., Bjarnadottir, K., Ingvarsson, R.F., et al. (2020). Humoral Immune Response to SARS-CoV-2 in Iceland. N. Engl. J. Med. *383*, 1724–1734.

Gunn, B.M., Lu, R., Slein, M.D., Ilinykh, P.A., Huang, K., Atyeo, C., Schendel, S.L., Kim, J., Cain, C., Roy, V., et al. (2021). A Fc engineering approach to define functional humoral correlates of immunity against Ebola virus. Immunity *54*, 815–828.

Hassan, A.O., Case, J.B., Winkler, E.S., Thackray, L.B., Kafai, N.M., Bailey, A.L., McCune, B.T., Fox, J.M., Chen, R.E., Alsoussi, W.B., et al. (2020a). A SARS-CoV-2 Infection Model in Mice Demonstrates Protection by Neutralizing Antibodies. Cell *182*, 744–753.

Hassan, A.O., Kafai, N.M., Dmitriev, I.P., Fox, J.M., Smith, B.K., Harvey, I.B., Chen, R.E., Winkler, E.S., Wessel, A.W., Case, J.B., et al. (2020b). A Single-Dose Intranasal ChAd Vaccine Protects Upper and Lower Respiratory Tracts against SARS-CoV-2. Cell *183*, 169–184.

Hassan, A.O., Feldmann, F., Zhao, H., Curiel, D.T., Okumura, A., Tang-Huau, T.L., Case, J.B., Meade-White, K., Callison, J., Lovaglio, J., et al. (2021). A single intranasal dose of chimpanzee adenovirus-vectored vaccine protects against SARS-CoV-2 infection in rhesus macaques. Cell Rep. Med, Published March 17, 2021. https://doi.org/10.1016/j.xcrm.2021.100230.

Hennrich, A.A., Sawatsky, B., Santos-Mandujano, R., Banda, D.H., Oberhuber, M., Schopf, A., Pfaffinger, V., Wittwer, K., Riedel, C., Pfaller, C.K., and Conzelmann, K.K. (2021). Safe and effective two-in-one replicon-and-VLP minispike vaccine for COVID-19: Protection of mice after a single immunization. PLoS Pathog. *17*, e1009064.

Hoffmann, M., Kleine-Weber, H., Schroeder, S., Krüger, N., Herrler, T., Erichsen, S., Schiergens, T.S., Herrler, G., Wu, N.H., Nitsche, A., et al. (2020). SARS-CoV-2 Cell Entry Depends on ACE2 and TMPRSS2 and Is Blocked by a Clinically Proven Protease Inhibitor. Cell *181*, 271–280.

Hsieh, C.L., Goldsmith, J.A., Schaub, J.M., DiVenere, A.M., Kuo, H.C., Javanmardi, K., Le, K.C., Wrapp, D., Lee, A.G., Liu, Y., et al. (2020). Structure-based design of prefusion-stabilized SARS-CoV-2 spikes. Science *369*, 1501–1505.

Letko, M., Marzi, A., and Munster, V. (2020). Functional assessment of cell entry and receptor usage for SARS-CoV-2 and other lineage B betacoronaviruses. Nat. Microbiol. *5*, 562–569.

Liu, Z., VanBlargan, L.A., Bloyet, L.M., Rothlauf, P.W., Chen, R.E., Stumpf, S., Zhao, H., Errico, J.M., Theel, E.S., Liebeskind, M.J., et al. (2021). Identification of SARS-CoV-2 spike mutations that attenuate monoclonal and serum antibody neutralization. Cell Host Microbe *29*, 477–488.

Madhi, S.A., Baillie, V., Cutland, C.L., Voysey, M., Koen, A.L., Fairlie, L., Padayachee, S.D., Dheda, K., Barnabas, S.L., Bhorat, Q.E., et al. (2021). Efficacy of the ChAdOx1 nCoV-19 Covid-19 Vaccine against the B.1.351 Variant. N. Engl. J. Med. *384*, 1885–1898.

Maizel, J.V., Jr., White, D.O., and Scharff, M.D. (1968). The polypeptides of adenovirus. I. Evidence for multiple protein components in the virion and a comparison of types 2, 7A, and 12. Virology *36*, 115–125.

Mao, R., Qiu, Y., He, J.S., Tan, J.Y., Li, X.H., Liang, J., Shen, J., Zhu, L.R., Chen, Y., Iacucci, M., et al. (2020). Manifestations and prognosis of gastrointestinal and liver involvement in patients with COVID-19: a systematic review and meta-analysis. Lancet Gastroenterol. Hepatol. *5*, 667–678.

McCallum, M., Bassi, J., Marco, A., Chen, A., Walls, A.C., Iulio, J.D., Tortorici, M.A., Navarro, M.J., Silacci-Fregni, C., Saliba, C., et al. (2021). SARS-CoV-2 immune evasion by variant B.1.427/B.1.429. Science, Published online July 1, 2021. https://doi.org/10.1126/science.abi7994.

Mercado, N.B., Zahn, R., Wegmann, F., Loos, C., Chandrashekar, A., Yu, J., Liu, J., Peter, L., McMahan, K., Tostanoski, L.H., et al. (2020). Single-shot Ad26 vaccine protects against SARS-CoV-2 in rhesus macaques. Nature *586*, 583–588.

Muñoz-Fontela, C., Dowling, W.E., Funnell, S.G.P., Gsell, P.S., Riveros-Balta, A.X., Albrecht, R.A., Andersen, H., Baric, R.S., Carroll, M.W., Cavaleri, M., et al. (2020). Animal models for COVID-19. Nature *586*, 509–515.

Pinto, D., Park, Y.J., Beltramello, M., Walls, A.C., Tortorici, M.A., Bianchi, S., Jaconi, S., Culap, K., Zatta, F., De Marco, A., et al. (2020). Cross-neutralization of SARS-CoV-2 by a human monoclonal SARS-CoV antibody. Nature *583*, 290–295.

Polack, F.P., Thomas, S.J., Kitchin, N., Absalon, J., Gurtman, A., Lockhart, S., Perez, J.L., Pérez Marc, G., Moreira, E.D., Zerbini, C., et al.; C4591001 Clinical Trial Group (2020). Safety and Efficacy of the BNT162b2 mRNA Covid-19 Vaccine. N. Engl. J. Med. *383*, 2603–2615.

Purtha, W.E., Tedder, T.F., Johnson, S., Bhattacharya, D., and Diamond, M.S. (2011). Memory B cells, but not long-lived plasma cells, possess antigen specificities for viral escape mutants. J. Exp. Med. *208*, 2599–2606.

Roy, S., Medina-Jaszek, A., Wilson, M.J., Sandhu, A., Calcedo, R., Lin, J., and Wilson, J.M. (2011). Creation of a panel of vectors based on ape adenovirus isolates. J. Gene Med. *13*, 17–25.

Sadoff, J., Le Gars, M., Shukarev, G., Heerwegh, D., Truyers, C., de Groot, A.M., Stoop, J., Tete, S., Van Damme, W., Leroux-Roels, I., et al. (2021). Interim Results of a Phase 1-2a Trial of Ad26.COV2.S Covid-19 Vaccine. N. Engl. J. Med *384*, 1824–1835.

Schäfer, A., Muecksch, F., Lorenzi, J.C.C., Leist, S.R., Cipolla, M., Bournazos, S., Schmidt, F., Maison, R.M., Gazumyan, A., Martinez, D.R., et al. (2021). Antibody potency, effector function, and combinations in protection and therapy for SARS-CoV-2 infection in vivo. J. Exp. Med. *218*, e20201993.

Sheehan, K.C., Lai, K.S., Dunn, G.P., Bruce, A.T., Diamond, M.S., Heutel, J.D., Dungo-Arthur, C., Carrero, J.A., White, J.M., Hertzog, P.J., and Schreiber, R.D. (2006). Blocking monoclonal antibodies specific for mouse IFN-alpha/beta receptor subunit 1 (IFNAR-1) from mice immunized by in vivo hydrodynamic transfection. J. Interferon Cytokine Res. *26*, 804–819.

Sun, S.H., Chen, Q., Gu, H.J., Yang, G., Wang, Y.X., Huang, X.Y., Liu, S.S., Zhang, N.N., Li, X.F., Xiong, R., et al. (2020). A Mouse Model of SARS-CoV-2 Infection and Pathogenesis. Cell Host Microbe. *28*, 124–133.

Tada, T., Dcosta, B.M., Samanovic-Golden, M., Herati, R.S., Cornelius, A., Mulligan, M.J., and Landau, N.R. (2021). Neutralization of viruses with European, South African, and United States SARS-CoV-2 variant spike proteins by convalescent sera and BNT162b2 mRNA vaccine-elicited antibodies. bioRxiv, Published online February 7, 2021. https://doi.org/10.1101/2021.02.05.430003.

Tortorici, M.A., Beltramello, M., Lempp, F.A., Pinto, D., Dang, H.V., Rosen, L.E., McCallum, M., Bowen, J., Minola, A., Jaconi, S., et al. (2020). Ultrapotent human antibodies protect against SARS-CoV-2 challenge via multiple mechanisms. Science *370*, 950–957.

Tostanoski, L.H., Wegmann, F., Martinot, A.J., Loos, C., McMahan, K., Mercado, N.B., Yu, J., Chan, C.N., Bondoc, S., Starke, C.E., et al. (2020). Ad26 vaccine protects against SARS-CoV-2 severe clinical disease in hamsters. Nat. Med. *26*, 1694–1700.

van Doremalen, N., Lambe, T., Spencer, A., Belij-Rammerstorfer, S., Purushotham, J.N., Port, J.R., Avanzato, V.A., Bushmaker, T., Flaxman, A., Ulaszewska, M., et al. (2020). ChAdOx1 nCoV-19 vaccine prevents SARS-CoV-2 pneumonia in rhesus macaques. Nature *586*, 578–582.

Vogel, A.B., Kanevsky, I., Che, Y., Swanson, K.A., Muik, A., Vormehr, M., Kranz, L.M., Walzer, K.C., Hein, S., Güler, A., et al. (2021). BNT162b vaccines protect rhesus macaques from SARS-CoV-2. Nature *592*, 283–289.

Wang, H., Zhang, Y., Huang, B., Deng, W., Quan, Y., Wang, W., Xu, W., Zhao, Y., Li, N., Zhang, J., et al. (2020). Development of an Inactivated Vaccine Candidate, BBIBP-CorV, with Potent Protection against SARS-CoV-2. Cell *182*, 713–721.

Wang, P., Nair, M.S., Liu, L., Iketani, S., Luo, Y., Guo, Y., Wang, M., Yu, J., Zhang, B., Kwong, P.D., et al. (2021a). Antibody Resistance of SARS-CoV-2 Variants B.1.351 and B.1.1.7. Nature *593*, 130–135.

Wang, Z., Schmidt, F., Weisblum, Y., Muecksch, F., Barnes, C.O., Finkin, S., Schaefer-Babajew, D., Cipolla, M., Gaebler, C., Lieberman, J.A., et al. (2021b). mRNA vaccine-elicited antibodies to SARS-CoV-2 and circulating variants. Nature *592*, 616–622.

Wessel, A.W., Kose, N., Bombardi, R.G., Roy, V., Chantima, W., Mongkolsapaya, J., Edeling, M.A., Nelson, C.A., Bosch, I., Alter, G., et al. (2020). Antibodies targeting epitopes on the cell-surface form of NS1 protect against Zika virus infection during pregnancy. Nat. Commun. *11*, 5278.

Wibmer, C.K., Ayres, F., Hermanus, T., Madzivhandila, M., Kgagudi, P., Lambson, B.E., Vermeulen, M., van den Berg, K., Rossouw, T., Boswell, M., et al. (2021). SARS-CoV-2 501Y.V2 escapes neutralization by South African COVID-19 donor plasma. Nat. Med *27*, 622–625.

Wichmann, D., Sperhake, J.P., Lütgehetmann, M., Steurer, S., Edler, C., Heinemann, A., Heinrich, F., Mushumba, H., Kniep, I., Schröder, A.S., et al. (2020). Autopsy Findings and Venous Thromboembolism in Patients With COVID-19: A Prospective Cohort Study. Ann. Intern. Med. *173*, 268–277.

Widge, A.T., Rouphael, N.G., Jackson, L.A., Anderson, E.J., Roberts, P.C., Makhene, M., Chappell, J.D., Denison, M.R., Stevens, L.J., Pruijssers, A.J., et al.; mRNA-1273 Study Group (2021). Durability of Responses after SARS-CoV-2 mRNA-1273 Vaccination. N. Engl. J. Med. *384*, 80–82.

Winkler, E.S., Bailey, A.L., Kafai, N.M., Nair, S., McCune, B.T., Yu, J., Fox, J.M., Chen, R.E., Earnest, J.T., Keeler, S.P., et al. (2020). SARS-CoV-2 infection of human ACE2-transgenic mice causes severe lung inflammation and impaired function. Nat. Immunol. *21*, 1327–1335.

Winkler, E.S., Gilchuk, P., Yu, J., Bailey, A.L., Chen, R.E., Chong, Z., Zost, S.J., Jang, H., Huang, Y., Allen, J.D., et al. (2021). Human neutralizing antibodies against SARS-CoV-2 require intact Fc effector functions for optimal therapeutic protection. Cell *184*, 1804–1820.

Wrapp, D., Wang, N., Corbett, K.S., Goldsmith, J.A., Hsieh, C.L., Abiona, O., Graham, B.S., and McLellan, J.S. (2020). Cryo-EM structure of the 2019-nCoV spike in the prefusion conformation. Science *367*, 1260–1263.

Xie, X., Muruato, A., Lokugamage, K.G., Narayanan, K., Zhang, X., Zou, J., Liu, J., Schindewolf, C., Bopp, N.E., Aguilar, P.V., et al. (2020). An Infectious cDNA Clone of SARS-CoV-2. Cell Host Microbe *27*, 841–848.

Yao, Y.F., Wang, Z.J., Jiang, R.D., Hu, X., Zhang, H.J., Zhou, Y.W., Gao, G., Chen, Y., Peng, Y., Liu, M.Q., et al. (2021). Protective Efficacy of Inactivated Vaccine against SARS-CoV-2 Infection in Mice and Non-Human Primates. Virol. Sin. Published online April 9, 2021. https://doi.org/10.1007/s12250-021-00376-w.

Yinda, C.K., Port, J.R., Bushmaker, T., Offei Owusu, I., Purushotham, J.N., Avanzato, V.A., Fischer, R.J., Schulz, J.E., Holbrook, M.G., Hebner, M.J., et al. (2021). K18-hACE2 mice develop respiratory disease resembling severe COVID-19. PLoS Pathog. *17*, e1009195.

Yu, J., Tostanoski, L.H., Peter, L., Mercado, N.B., McMahan, K., Mahrokhian, S.H., Nkolola, J.P., Liu, J., Li, Z., Chandrashekar, A., et al. (2020). DNA vaccine protection against SARS-CoV-2 in rhesus macaques. Science *369*, 806–811.

Zang, R., Gomez Castro, M.F., McCune, B.T., Zeng, Q., Rothlauf, P.W., Sonnek, N.M., Liu, Z., Brulois, K.F., Wang, X., Greenberg, H.B., et al. (2020). TMPRSS2 and TMPRSS4 promote SARS-CoV-2 infection of human small intestinal enterocytes. Sci. Immunol. *5*, eabc3582.

Zhou, F., Yu, T., Du, R., Fan, G., Liu, Y., Liu, Z., Xiang, J., Wang, Y., Song, B., Gu, X., et al. (2020). Clinical course and risk factors for mortality of adult inpatients with COVID-19 in Wuhan, China: a retrospective cohort study. Lancet *395*, 1054–1062.

Zhou, D., Dejnirattisai, W., Supasa, P., Liu, C., Mentzer, A.J., Ginn, H.M., Zhao, Y., Duyvesteyn, H.M.E., Tuekprakhon, A., Nutalai, R., et al. (2021). Evidence of escape of SARS-CoV-2 variant B.1.351 from natural and vaccine-induced sera. Cell *184*, 2348–2361.

Zost, S.J., Gilchuk, P., Chen, R.E., Case, J.B., Reidy, J.X., Trivette, A., Nargi, R.S., Sutton, R.E., Suryadevara, N., Chen, E.C., et al. (2020). Rapid isolation and profiling of a diverse panel of human monoclonal antibodies targeting the SARS-CoV-2 spike protein. Nat. Med. *26*, 1422–1427.

CellPress
OPEN ACCESS

STAR★METHODS

KEY RESOURCES TABLE

Reagent or resource	Source	Identifier
**Antibodies**		
Anti-SARS-CoV-2 mAb cocktail	Liu et al., 2021	N/A
Goat anti-mouse IgG-HRP	Southern Biotech	Cat # 1030-05; RRID:AB_2619742
Goat anti-mouse IgA-HRP	Southern Biotech	Cat # 1040-05; RRID:AB_2714213
PE-anti CD138	BD BioSciences	553714; RRID:AB_395000
MAR1-5A3, anti-Ifnar1 mAb	Leinco	I-401; RRID:AB_2491621
Steptavidin-R-Phycoerythrin	Prozyme	PJ31S
IgG2a	Southern Biotech	Cat # 1080-09S; RRID:AB_2794481
IgG2b	Southern Biotech	Cat # 1090-09S; RRID:AB_2794524
IgG3	Southern Biotech	Cat # 1100-09; RRID:AB_2794576
IgM	Southern Biotech	Cat # 1020-09; RRID:AB_2794204
IgA	Southern Biotech	Cat # 1040-09; RRID:AB_2794375
CD11b APC	Biolegend	Cat # 101212; RRID:AB_312795
Ly6G Pacific Blue	Biolegend	Cat # 127628; RRID:AB_2562567
Ly6C Brilliant Violet 605	Biolegend	Cat # 128036; RRID:AB_2562353
Fc Block	BD Biosciences	Cat # 553142; RRID:AB_394657
IgG1	Southern Biotech	Cat # 1070-09; RRID:AB_2794414
CD11c AlexaFluro700	BioLegend	117320; RRID:AB_528736
**Virus and bacterial strains**		
SARS-CoV-2 (strain 2019 n-CoV/ USA_WA1/2020)	CDC/BEI Resources	NR52281
SARS-CoV-2 (Recombinant strain: Wash-B.1.1.351)	Chen et al., 2021	N/A
SASR-CoV-2 (Recombinant strain: Wash-B.1.1.28)	Chen et al., 2021	N/A
SASR-CoV-2 (Strain B.1.267.1)	Dr. Mehul Suthar and Dr. Pei-Yong Shi	N/A
ChAd-SARS-CoV-2-S	Hassan et al., 2020b	N/A
ChAd-Control	Hassan et al., 2020b	N/A
AdV5-hACE2	Hassan et al., 2020b	N/A
**Experimental models: Cell lines**		
Vero CCL-81	ATCC	CCL-81; RRID: CVCL_0059
Vero E6	ATCC	CRL-1586; RRID:CVCL_0574
Vero TMPRSS2	ATCC	RRID:CVCL_YQ48
J774A.1	ATCC	TIB-67; RRID:CVCL_0358
**Experimental models: Organisms/strains**		
Mouse: BALB/c	Jackson Laboratory	Cat#000651; RRID: IMSR_JAX:000651
Mouse: B6.Cg-Tg(K18-ACE2)2Prlmn/J	Jackson Laboratory	Cat# 034860; RRID:IMSR_JAX:034860
**Chemicals, peptides, recombinant proteins**		
EDC	Thermo Fisher Scientific	A35391
Sulfo-NHS	Thermo Fisher Scientific	A39269
SARS-CoV-2 Spike	Dr. Daved Fremont and Dr. Erica Saphire	N/A
SARS-CoV-2 RBD	Dr. Daved Fremont and Dr. Florian Krammer	N/A
Murine FcγRIIb, RIII, RIV	Produced at the Duke Human Vaccine Institute	N/A

(*Continued on next page*)

Continued		
Reagent or resource	Source	Identifier
Oligonucleotides		
SARS-CoV-2 N F: 5′-ATGCT GCAATCGTGCTACAA-3′	Hassan et al., 2020a	N/A
SARS-CoV-2 N R: 5′-GACTG CCGCCTCTGCTC-3′	Hassan et al., 2020a	N/A
SARS-CoV-2 N Probe: 5′-/56-FAM/ TCAAGGAAC/ZEN/AACATTGC CAA/3IABkFQ/-3′	Hassan et al., 2020a	N/A
Software and algorithms		
FlowJo	FlowJo, LLC	v10
GraphPad Prism	GraphPad	v 8.2.1
Biorender	biorender.com	N/A
Jupyter Notebook	Jupyter	6.1.4
Other		
EasySep Mouse CD138 Positive Selection	STEMCELL	Cat # 18957
Red FluoSphereTM Carboxylate-Modified Microsphere, 0.2 μ M	ThermoFisher Scientific	F8810
Yellow-green FluoSphereTM Carboxylate-Modified Microsphere, 0.2 μ M	ThermoFisher Scientific	F8811
Blue FluoSphereTM Carboxylate-Modified Microsphere, 0.2 μ M	ThermoFisher Scientific	F8805
MagPlex microsphere	Luminex corporation	MC12003-01, MC10019-YY, MC10020-YY, MC10021-YY, MC10033-YY, MC10035-YY, MC10042-YY, MC10077-YY, MC12004-01, MC12001-01, MC12005-01

RESOURCE AVAILABILITY

Lead contact

Further information and requests for resources and reagents should be directed to and will be fulfilled by the Lead Contact, Michael S. Diamond (diamond@wusm.wustl.edu).

Materials availability

All requests for resources and reagents should be directed to and will be fulfilled by the Lead Contact author. This includes mice, antibodies, viruses, vaccines, and proteins. All reagents will be made available on request after completion of a Materials Transfer Agreement.

Data and code availability

All data supporting the findings of this study are available within the paper or from the corresponding author upon request. This paper does not report original code. Any additional information required to reanalyze the data reported in this paper is available from the lead contact upon request.

EXPERIMENTAL MODEL AND SUBJECT DETAILS

Viruses and cells

Vero E6 (CRL-1586, American Type Culture Collection (ATCC), Vero-TMPRSS2 (Zang et al., 2020), Vero (CCL-81, ATCC) and HEK293 (CRL-1573, ATCC) cells were cultured at 37°C in Dulbecco's Modified Eagle medium (DMEM) supplemented with 10% fetal bovine serum (FBS), 10 mM HEPES pH 7.3, 1 mM sodium pyruvate, 1X non-essential amino acids, and 100 U/ml of penicillin–streptomycin. Vero-TMPRSS2 cells also were supplemented with 5 μg/mL of blasticidin.

SARS-CoV-2 strain 2019n-CoV/USA_WA1/2020 (WA1/2020) was obtained from the Centers for Disease Control and Prevention. The virus was passaged once in Vero CCL-81 cells and titrated by focus-forming assay (FFA) on Vero E6 cells. The Wash-B.1.351 and Wash-B.1.1.28 chimeric viruses with variant spike genes were described previously (Chen et al., 2021; Xie et al., 2020), passaged

once in Vero-TMPRSS2 cells, and subjected to next-generation sequencing to confirm the introduction and stability of substitutions. The B.1.617.1 variant was plaque purified from a midturbinate nasal swab, passaged twice on Vero-TMPRSS2 cells, and next-generation sequenced (spike substuitutions: G142D, E154K, L452R, E484Q, D614G, P681R, Q1071H, and H1101D). All virus experiments were performed in an approved Biosafety level 3 (BSL-3) facility.

Mouse experiments

Animal studies were carried out in accordance with the recommendations in the Guide for the Care and Use of Laboratory Animals of the National Institutes of Health. The protocols were approved by the Institutional Animal Care and Use Committee at the Washington University School of Medicine (Assurance number A3381-01). Virus inoculations were performed under anesthesia that was induced and maintained with ketamine hydrochloride and xylazine, and all efforts were made to minimize animal suffering.

Female BALB/c (catalog 000651) and K18-hACE2 C57BL/6 (catalog 034860) mice were purchased from The Jackson Laboratory. Four to five-week-old animals were immunized with 10^{10} vp of ChAdV-control or 10^8, 10^9, or 10^{10} vp of ChAd-SARS-CoV-2-S in 50 μl PBS via IM (hind leg) or IN injection. Vaccinated BALB/c mice (10 to 11-week-old) were given a single intraperitoneal injection of 2 mg of anti-Ifnar1 mAb (MAR1-5A3 (Sheehan et al., 2006) (Leinco) one day before IN administration of 10^8 PFU of Hu-Ad5-hACE2 (Hassan et al., 2020a). Five days after Hu-Ad5–hACE2 transduction, mice were inoculated with 4×10^5 FFU of WA1/2020 SARS-CoV-2 by the IN route. K18-hACE2 mice were challenged on indicated days after immunization with 10^4 FFU of SARS-CoV-2 (WA1/2020, Wash-B.1.351, or Wash-B.1.1.28) via IN route. Animals were euthanized at 6 dpi, and tissues were harvested for virological analysis.

METHOD DETAILS

Chimpanzee and human adenovirus vectors

The ChAd-SARS-CoV-2 and ChAd-Control vaccine vectors were derived from simian Ad36 backbones (Roy et al., 2011), and the constructing and validation has been described in detail previously (Hassan et al., 2020b). The rescued replication-incompetent ChAd-SARS-CoV-2-S and ChAd-Control vectors were scaled up in HEK293 cells and purified by CsCl density-gradient ultracentrifugation. Viral particle concentration in each vector preparation was determined by spectrophotometry at 260 nm as described (Maizel et al., 1968). The Hu-AdV5-hACE2 vector also was described previously (Hassan et al., 2020b) and produced in HEK293 cells. The viral titer was determined by plaque assay in HEK293 cells.

SARS-CoV-2 neutralization assays

Heat-inactivated serum samples were diluted serially and incubated with 10^2 FFU of different SARS-CoV-2 strains for 1 h at 37°C. The virus-serum mixtures were added to Vero cell monolayers in 96-well plates and incubated for 1 h at 37°C. Subsequently, cells were overlaid with 1% (w/v) methylcellulose in MEM supplemented with 2% FBS. Plates were incubated for 30 h before fixation using 4% PFA in PBS for 1 h at room temperature. Cells were washed and then sequentially incubated with an oligoclonal pool of SARS2-2, SARS2-11, SARS2-16, SARS2-31, SARS2-38, SARS2-57, and SARS2-71 (Liu et al., 2021) anti-S antibodies and HRP-conjugated goat anti-mouse IgG (Sigma, 12-349) in PBS supplemented with 0.1% saponin and 0.1% bovine serum albumin. TrueBlue peroxidase substrate (KPL) was used to develop the plates before counting the foci on a BioSpot analyzer (Cellular Technology Limited).

Protein expression and purification

The cloning and production of purified S and RBD proteins corresponding to the WA1/2020 SARS-CoV-2 strain have been described previously (Alsoussi et al., 2020; Hassan et al., 2020b). Briefly, prefusion-stabilized S (Hsieh et al., 2020) and RBD were cloned into a pCAGGS mammalian expression vector with a hexahistidine tag and transiently transfected into Expi293F cells. Proteins were purified by cobalt-charged resin chromatography (G-Biosciences).

ELISA

Purified antigens (S or RBD) were coated onto 96-well Maxisorp clear plates at 2 μg/mL in 50 mM Na_2CO_3 pH 9.6 (70 μL) overnight at 4°C. Coating buffers were aspirated, and wells were blocked with 200 μL of 1X PBS + 0.05% Tween-20 + 1% BSA + 0.02% NaN_3 (Blocking buffer, PBSTBA) overnight at 4°C. Heat-inactivated serum samples were diluted in PBSTBA in a separate 96-well polypropylene plate. The plates then were washed thrice with 1X PBS + 0.05% Tween-20 (PBST), followed by addition of 50 μL of respective serum dilutions. Sera were incubated in the blocked ELISA plates for at least 1 h at room temperature. The ELISA plates were again washed thrice in PBST, followed by addition of 50 μL of 1:1,000 anti-mouse IgG-HRP (Southern Biotech Cat. #1030-05) in PBST or 1:1000 of anti-mouse IgA-HRP in PBSTBA (SouthernBiotech). Plates were incubated at room temperature for 1 h, washed thrice in PBST, and then 100 μL of 1-Step Ultra TMB-ELISA was added (ThermoFisher Cat. #34028). Following a 10 to 12-min incubation, reactions were stopped with 50 μL of 2 M sulfuric acid. Optical density (450 nm) measurements were determined using a microplate reader (Bio-Rad).

ELISPOT assay

To quantitate S-specific plasma cells in the bone marrow, femurs and tibias were crushed using a mortar and pestle in RPMI 1640, filtered through a 100 μm strainer and subjected to ACK lysis. $CD138^+$ cells were enriched by positive selection and magnetic beads

according to the manufacturer's instructions (EasySep Mouse CD138 Positive Selection, STEMCELL). The enriched $CD138^+$ cells were incubated overnight in RPMI 1640 supplemented with 10% FBS in MultiScreen-HA Filter Plates (Millipore) pre-coated with SARS-CoV-2 S protein. Foci were developed using TruBlue substrate (KPL) following sequential incubation with anti-mouse IgG-biotin or anti-mouse IgA-biotin and streptavidin-HRP. Plates were imaged using a BioSpot instrument, and foci enumerated manually.

Measurement of viral burden

SARS-CoV-2 infected mice were euthanized using a ketamine and xylazine cocktail, and organs were collected. Tissues were weighed and homogenized with beads using a MagNA Lyser (Roche) in 1 mL of Dulbecco's Modified Eagle's Medium (DMEM) containing 2% fetal bovine serum (FBS). RNA was extracted from clarified tissue homogenates using MagMax mirVana Total RNA isolation kit (Thermo Scientific) and the KingFisher Flex extraction system (Thermo Scientific). SARS-CoV-2 RNA levels were measured by one-step quantitative reverse transcriptase PCR (qRT-PCR) TaqMan assay as described previously (Hassan et al., 2020a). SARS-CoV-2 nucleocapsid (N) specific primers and probe sets were used: (N: F Primer: ATGCTGCAATCGTGCTACAA; R primer: GACTGCCGCCTCTGCTC; probe: /56-FAM/TCAAGGAAC/ZEN/AACATTGCCAA/3IABkFQ) (Integrated DNA Technologies). Viral RNA was expressed as (N) gene copy numbers per milligram on a log_{10} scale.

Luminex analysis

Luminex analhysis was conducted as described previously (Brown et al., 2017). Briefly, proteins (Spike: D614G, E484K, N501Δ69-70, K417N, B.1.1.7, B.1.351; RBD (ImmuneTech): WT, E484K, B.1.1.7, B.1.351, B.1.128) were carboxy-coupled to magnetic Luminex microplex carboxylated beads (Luminex Corporation) using NHS-ester linkages with Sulfo-NHS and EDC (Thermo Fisher) and then incubated with serum (IgG1, FcγRIIb, FcγRIII 1:3000; IgG2a, G2b, G3, A, FcγRIV 1:1000, IgM 1:500) for 2 h at 37°C. Isotype analysis was perfomed by incubating the immune complexes with secondary goat anti-mouse-PE antibody (IgG1 1070-09, IgG2a 1080-09S, IgG2b 1090-09S, IgG3 1100-09, IgM 1020-09, IgA 1040-09 Southern Biotech) for each isotype. FcγR binding was quantified by incubating immune complexes with biotinylated FcγRs (FcγRIIB, FcγRIII, and FcγRIV, courtesy of Duke Protein Production Facility) conjugated to Steptavidin-PE (Prozyme). Flow cytometry was performed with an IQue (Intellicyt), and analysis was performed on IntelliCyt ForeCyt (v8.1).

Antibody-dependent neutrophil or cellular phagocytosis

Antibody-dependent neutrophil phagocytosis (ADNP) and cellular phagocytosis (ADCP) assays were conducted as described previously (Butler et al., 2019; Gunn et al., 2021; Wessel et al., 2020). Briefly, spike protein was carboxy coupled to blue, yellow-green, or red FluoSphere™ Carboxylate-modified microsphere, 0.2 μm (ThermoFisher) using NHS-ester linkages with Sulfo-NHS and EDC (Thermo Fisher). Spike-coated beads were incubated with diluted serum (1:150 ADNP, 1:100 ADCP) for 2 hours at 37°C. For the ADNP assay, bone marrow cells were collected from BALB/c mice, and red blood cells were subjected to ACK lysis. The remaining cells were washed with PBS, and aliquoted into 96-well plates (5×10^4 cells per well). The bead-antibody complexes were added to cells and incubated for 1 h at 37°C. After washing, cells were stained with the following antibodies: CD11b APC (BioLegend 101212), CD11c A700 (BioLegend 117320), Ly6G Pacific Blue (127628), Ly6C BV605 (BioLegend 128036), Fcblock (BD Bioscience 553142) and CD3 PE/Cy7 (BioLegend 100320). Cells were fixed with 4% PFA, processed on an BD LSRFortessa (BD Biosciences). Neutrophils were defined as $CD3^-$, $CD11b^+$, $Ly6G^+$. The neutrophil phagocytosis score was calculated as (% FITC+) x (geometic mean fluorescent intensity of FITC)/10000. For the ADCP assay, J774A.1 (ATCC TIB-67) murine monocytic cells were incubated with the Spike-coated bead–antibody complexes for 1 h at 37°C. Cells were washed in 5 mM EDTA PBS, fixed with 4% PFA, and analyzed on an BD LSRFortessa (BD Biosciences). The cellular phagocytosis score was calculated as (% FITC+) x (geometic mean fluorescent intensity of FITC)/10000.

QUANTIFICATION AND STATISTICAL ANALYSIS

Statistical significance was assigned when *P value*s were < 0.05 using Prism Version 8 (GraphPad) or Jupyter Notebook 6.1.4. Tests, number of animals (n), median values, and statistical comparison groups are indicated in the Figure legends. Analysis of anti-S, anti-RBD, neutralization titers, and ELISPOT values after vaccination was performed using a one-way ANOVA with a Dunnett's post-test or a Mann-Whitney test. Differences in viral titers after SARS-CoV-2 infection of immunized mice were determined using a Kruskal Wallis ANOVA with Dunn's post-test or a Mann-Whitney test. Differences in neutralization titers for different variants were compared using two-tailed Wilcoxon matched-pairs signed rank test. Weight changes were analyzed using area under the curve analysis and a Student's t test.

Cell Reports, Volume 36

Supplemental information

An intranasal vaccine durably protects against SARS-CoV-2 variants in mice

Ahmed O. Hassan, Swathi Shrihari, Matthew J. Gorman, Baoling Ying, Dansu Yaun, Saravanan Raju, Rita E. Chen, Igor P. Dmitriev, Elena Kashentseva, Lucas J. Adams, Colin Mann, Meredith E. Davis-Gardner, Mehul S. Suthar, Pei-Yong Shi, Erica Ollmann Saphire, Daved H. Fremont, David T. Curiel, Galit Alter, and Michael S. Diamond

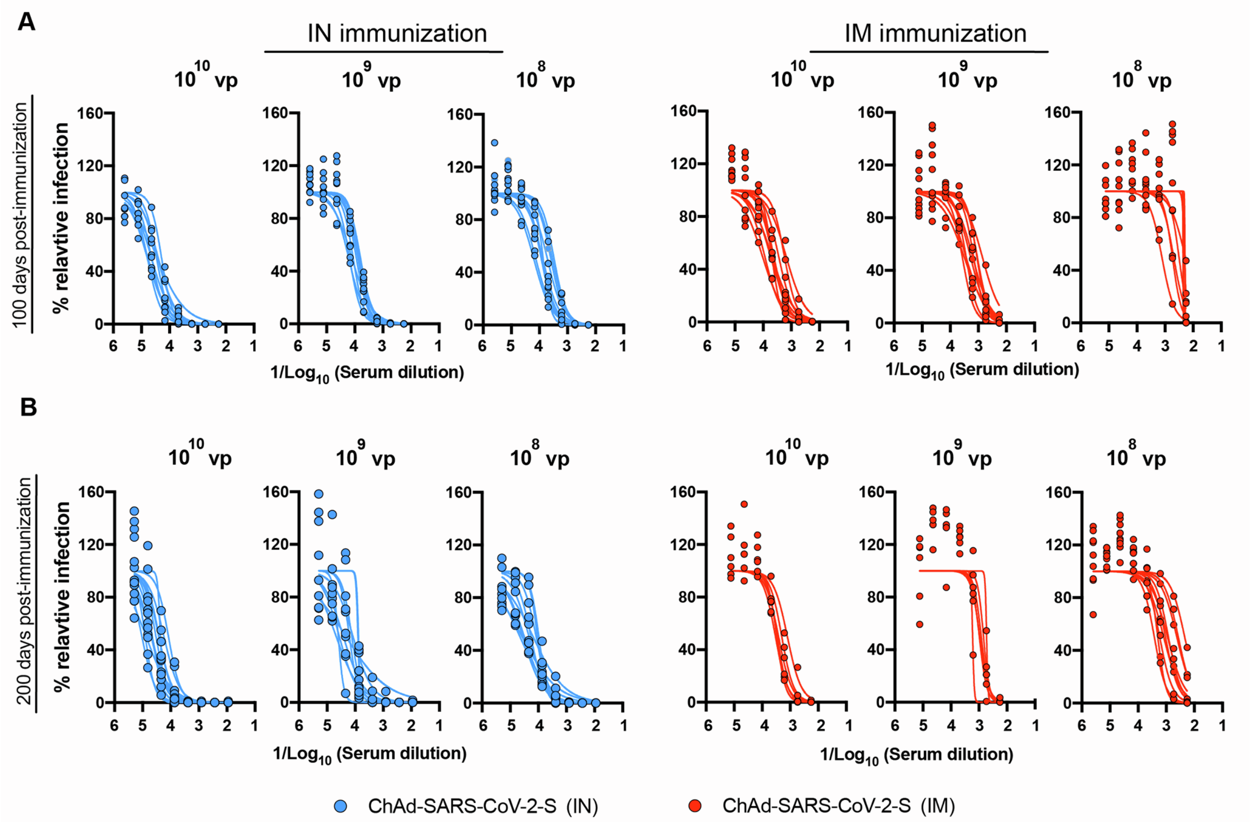

Figure S1. ChAd-SARS-CoV-2-S vaccine induces neutralizing antibodies as measured by FRNT. Related to Figure 1. Five-week old female BALB/c mice were immunized via IN or IM route with a single 10^{10}, 10^{9}, or 10^{8} dose of ChAd-SARS-CoV-2-S. Serum samples from ChAd-SARS-CoV-2-S vaccinated mice were collected at days 100 (**A**) or 200 (**B**) after immunization and assayed for neutralizing activity by FRNT. Serum neutralization curves corresponding to individual mice are shown for the indicated vaccines (n = 6-14 per group). Each point represents the mean of two technical replicates.

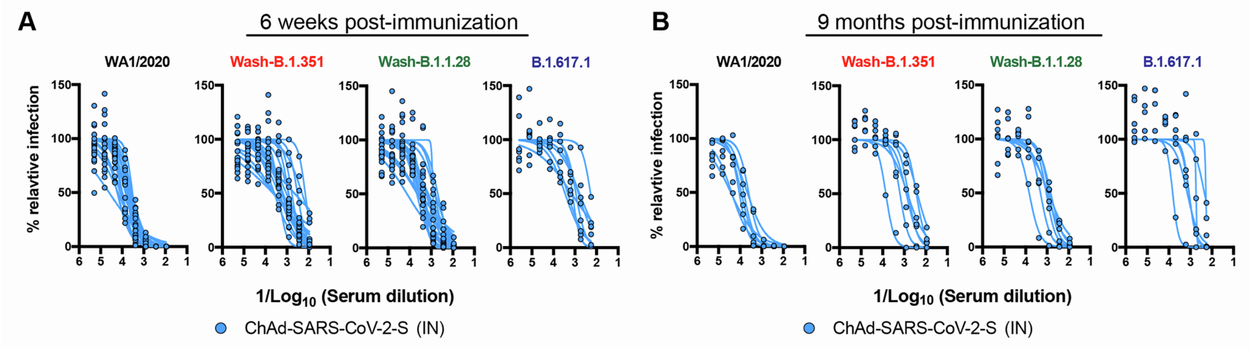

Figure S2. ChAd-SARS-CoV-2-S vaccine induces neutralizing antibodies as measured by FRNT. Related to Figure 4. Five-week-old K18-hACE2 female mice were immunized with 10^9 vp ChAd-control or ChAd-SARS-CoV-2-S via an IN route. Serum samples were collected at six weeks (**A**) or nine months (**B**) after immunization and assayed for neutralizing activity against WA1/2020, Wash-B.1.351, Wash-B.1.1.28, or B.1.617.1 by FRNT. Serum neutralization curves corresponding to individual mice are shown for the indicated vaccines (n = 7-20 per group). Each point represents the mean of two technical replicates.
